# ChMob2 binds to ChCbk1 and promotes virulence and conidiation of the fungal pathogen *Colletotrichum higginsianum*

**DOI:** 10.1186/s12866-017-0932-7

**Published:** 2017-01-19

**Authors:** Johannes Schmidpeter, Marlis Dahl, Jörg Hofmann, Christian Koch

**Affiliations:** Department of Biology, Division of Biochemistry, Friedrich-Alexander University Erlangen-Nuremberg, Staudtstrasse 5, 91058 Erlangen, Germany

**Keywords:** Phytopathogenic ascomycete fungus, ATMT, *Colletotrichum higginsianum*, Conidiation, NDR Kinase Cbk1, Mob1, Mob2, Mob3, Ace2, Ssd1, Cts1

## Abstract

**Background:**

Mob family proteins are conserved between animals, plants and fungi and are essential for the activation of NDR kinases that control crucial cellular processes like cytokinesis, proliferation and morphology.

**Results:**

We identified a hypomorphic allele of *ChMOB2* in a random insertional mutant (*vir-88*) of the hemibiotrophic ascomycete fungus *Colletotrichum higginsianum*. The mutant is impaired in conidiation, host penetration and virulence on *Arabidopsis thaliana*. ChMob2 binds to and co-localizes with the NDR/LATS kinase homolog ChCbk1. Mutants in the two potential ChCbk1 downstream targets *ChSSD1* and *ChACE2* show defects in pathogenicity. The genome of *C. higginsianum* encodes two more Mob proteins. While we could not detect any effect on pathogenicity in Δ*Chmob3* mutants, ChMob1 is involved in conidiation, septae formation and virulence.

**Conclusion:**

This study shows that ChMob2 binds to the conserved NDR/LATS Kinase ChCbk1 and plays an important role in pathogenicity of *Colletotrichum higginsianum* on *Arabidopsis thaliana*.

**Electronic supplementary material:**

The online version of this article (doi:10.1186/s12866-017-0932-7) contains supplementary material, which is available to authorized users.

## Background

Infection of host plants by filamentous, appressoria forming fungi like the ascomycete *Colletotrichum higginsianum* depends on directed, polarized growth and morphological switches of infection structures [1–5]. This fungus employs a hemibiotrophic infection strategy that includes two phases [6]. After an initial biotrophic phase with bulbous primary hyphae, *Colletotrichum higginsianum* switches to a necrotrophic growth phase forming characteristic thin secondary hyphae. During penetration by the appressorium, which itself is a morphologically highly differentiated cell [7], the tip of the penetration peg grows directed from the penetration pore towards the host epidermis [8, 9]. The infection vesicle and the primary hyphae grow out of this structure into the first host cell, which still has an intact plasma membrane and remains alive. After the switch to necrotrophy, secondary hyphae develop. These hyphae invade neighboring cells and grow strongly polarized at the hyphal tip similar to saprophytic hyphae of other filamentous fungi [10]. For such processes, the establishment and maintenance of polarity and cell wall morphogenesis are critical. In fungi, their regulation is influenced by pathways which include a central kinase and a Mob-family protein that acts as co-activator [11]. These pathways are called RAM (Regulation of Ace2p activity and morphogenesis) and MEN (Mitotic exit network) in *S. cerevisiae* [12, 13] or MOR (Morphogenesis-related) and SIN (Septum initiation network) in *S. pombe* [14, 15]*.* In *S. cerevisiae*, polarized growth, cell wall morphogenesis and other processes like cell separation, daughter cell-specific gene expression and cell cycle progression are influenced by the RAM pathway [12]. Yeast Cbk1 is the terminal kinase in this pathway and belongs to the nuclear Dbf2-related (NDR)/large tumor suppressor (LATS) kinase subfamily, which is conserved from yeast to man [11]. Mob2 binds to Cbk1 and is essential for Cbk1 kinase activity and its proper localization in yeast [16]. Until now, only some Cbk1 targets have been identified. E.g. in yeast, Cbk1 binds and phosphorylates Sec2, which is involved in polarized vesicle exocytosis [17], and Ssd1, a mRNA-binding protein that associates with many transcripts including chitinase *CTS1* mRNA [18]. Aside from polarized cell morphology, a well-established function of the RAM network in yeast is the control of activity and localization of the zinc finger transcription factor Ace2 during late mitosis [16] that regulates cell wall and cell separation genes like the chitinase *CTS1* and the glucanase *SCW11* specifically in the new daughter cell [19]. Nuclear localization of Ace2 is also coordinated with the MEN signaling pathway [20], which uses the NDR/LATS kinase Dbf2 and its associated kinase activator Mob1 [13].

The founding member of the NDR family, Cot1, is similar to yeast Cbk1 and was identified in the filamentous fungus *Neurospora crassa* as a temperature sensitive mutant allele (colonial temperature-sensitive 1) that showed impaired hyphal tip elongation [21]. Like its yeast homologs, Cot1 requires binding of a Mob co-activator protein for its activity. This function appears to be mediated by two Mob2 proteins in *N. crassa*, called Mob2a and Mob2b [22]. Filamentous fungi such as *N. crassa* and its close relative *S. macrospora* encode a third type of Mob-family protein, called Mob3 [22, 23]. Mob3 is more similar to the mammalian striatin-binding protein phocein, does not seem to be involved in NDR signalling but was found to be important for the development of protoperithecia and for hyphal fusion [22–24].

Previously, we described the identification of 75 *C. higginsianum Agrobacterium tumefaciens*-mediated transformation (ATMT) mutants, which are impaired in virulence on *Arabidopsis thaliana* [25]. Here, we show that for one of these mutants (*vir-88),* a hypomorphic allele of *ChMOB2* is responsible for the virulence phenotype and we analyzed the function of the protein encoded by this locus in *C. higginsianum*. We found that ChMob2 is required for both conidiation and formation of functional appressoria and that it binds to the NDR/LATS kinase ChCbk1. We searched for potential targets of the Mob2/Cbk1 complex and observed that mutants of two candidate genes have phenotypes similar to *vir-88*. Furthermore, we analyzed the roles of *ChMOB3* and *ChMOB1* in *C. higginsianum*.

## Results

### *vir-88* has defects in appressoria shape and production of conidia

**Fig. 1:**
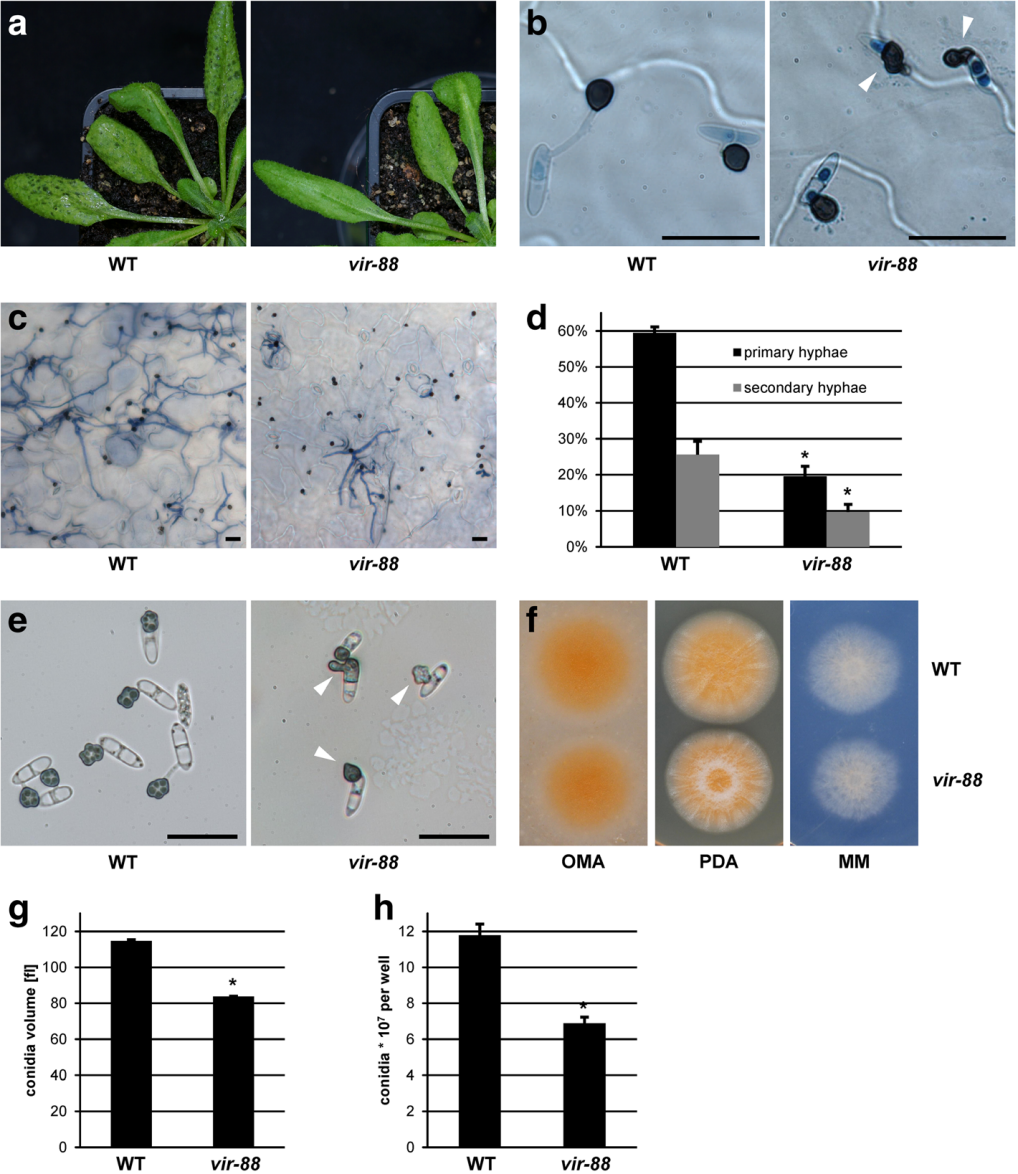
T-DNA insertion mutant *vir-88* is impaired in virulence and conidiation. *A. thaliana* Col-0 plants were infected with *C. higginsianum* WT and the *vir-88* mutant strain. **a** Macroscopic image at 4 dpi. **b** Microscopic images of germinated conidia with appressoria at 1 dpi on trypan blue stained leaves. Appressoria with unusual morphology are marked with white arrows. **c** Trypan blue stained *A. thaliana* leaves at 3 dpi. **d** Quantification of trypan blue stained primary hyphae (relative to appressoria) and secondary hyphae (relative to primary hyphae) at 3 dpi. **e** Appressoria of WT and *vir-88* on artificial hydrophobic surface. Appressoria with unusual morphology are marked with arrows. Scale bars = 20 μm. **f** Vegetative growth of WT and *vir-88* on oatmeal agar (OMA), potato dextrose agar (PDA) and Czapek-Dox minimal medium (MM). **g**, **h** Average conidial cell volume **g** and conidial titer **h** of WT and *vir-88* strains recovered from oatmeal agar after 7 days. The data of 3 separate plates is given. Error bars are represented as the standard error from three replicates. Significant differences based on t-tests (*p* < 0.05) are marked with asterisks

*vir-88* was isolated from a pool of T-DNA insertion mutants with defects in virulence on *Arabidopsis thaliana* [25]. In contrast to *Arabidopsis* infected with wild-type *C. higginsianum* where all leaves showed strong necrotic lesions after 4 days, infection with *vir-88* resulted only in small and much fewer lesions (Fig. 1a). Staining infected leaves with trypan blue revealed that mutant *vir-88* formed appressoria at levels comparable to wild-type, but they were often severely deformed (Fig. 1b, Additional file 1: Figure S1). At 3 dpi, nearly two thirds of the appressoria produced by *vir-88* showed morphological defects like elongation, decreased diameter, small lateral protrusions or were less melanized. Some cells formed a melanized germ tube or a partly melanized conidium with or without an appressorium. Examples of these morphological phenotypes are shown in Fig. 1b and Additional file 1: Figure S1A. In contrast, the wild-type produced only 11% altered appressoria with less dramatic alterations (Additional file 1: Figure S1B). The ability of morphologically abnormal appressoria to establish infectious primary hyphae was strongly reduced because only 10% of all primary hyphae emanated from abnormal appressoria with morphological defects (Additional file 1: Figure S1C). As a consequence, the amount of primary hyphae formed by *vir-88* at 3 dpi was reduced to only one third of the wild-type level (Fig. 1c, d). The fraction of primary hyphae that gave rise to secondary hyphae was reduced by 62% in *vir-88* (Fig. 1c, d). On artificial hydrophobic plastic surfaces, appressoria of *vir-88* were similarly deformed in 64% of the cases compared to 7% in the WT (Fig. 1e, Additional file 1: Figure S1D).

When inoculated on potato dextrose agar or minimal medium plates, *vir-88* produced slightly smaller colonies than the wild-type (Fig. 1f). Furthermore, *vir-8*8 did not show different sensitivity to several stress inducing compounds than the wild-type (Additional file 2: Figure S2). In addition to its virulence phenotype, mutant *vir-88* generated less (42%) conidia on oatmeal agar plates, which were significantly smaller than wild-type conidia (Fig. 1g, h). Taken together, the T-DNA insertion mutant *vir-88* is severely impaired in virulence due to defects during appressoria differentiation and host cell penetration.

### *vir-88* encodes a hypomorphic allele of *ChMOB2*

**Fig. 2:**
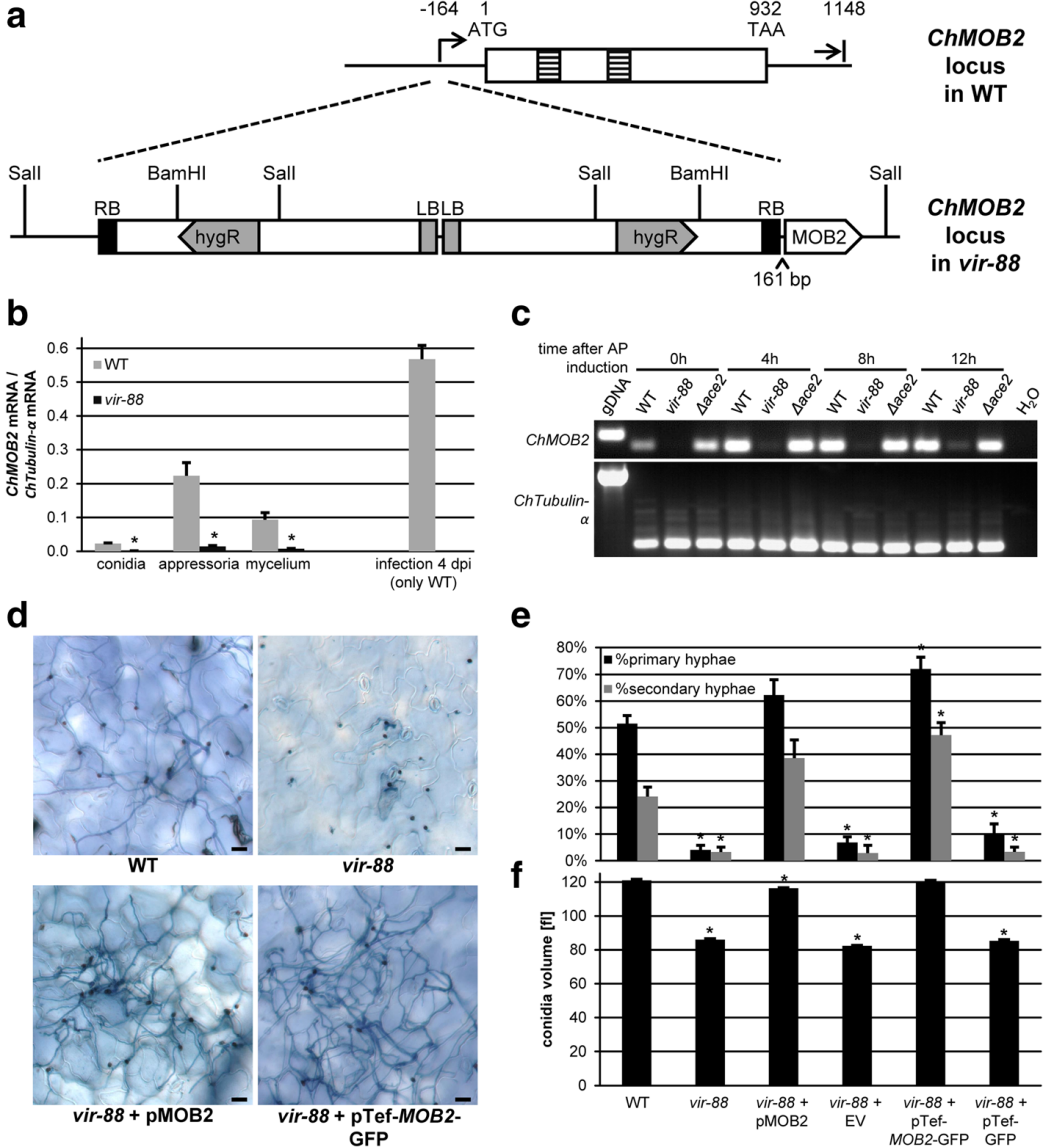
*vir-88* encodes a hypomorphic *ChMOB2* allele. **a** Schematic representation of the *ChMOB2* locus in wild-type (top) and *vir-88* (bottom). Beginning and end of the *ChMOB2* transcript are shown relative to the start codon. Introns are depicted as striped boxes. LB = left border; RB = right border. **b** Quantitative RT-PCR of *ChMOB2* mRNA abundance in conidia, appressoria, mycelium and in infected plants (4 dpi; in this case only data from WT is shown). The data were normalized against α-TUBULIN (CH063_01222) and are shown as the average of three biological replicates. **c** Semi-quantitative RT-PCR of *ChMOB2* and α-TUBULIN in RNA samples from in vitro appressoria 0, 4, 8 and 12 h after inoculation. cDNAs from WT, *vir-88* and Δ*Chace2* (CY6353, see below) strains were used as template for PCR. Genomic WT DNA (gDNA) or H_2_O were used as controls. **d**, **e** *A. thaliana* leaves at 3 days after infection with WT, *vir-88* and *vir-88* strains complemented with either the *ChMOB2* wild-type allele (pMOB2, pCK3110) or with a pTef-*MOB2*-GFP construct (pCK4129). Typical trypan blue stained leaves are shown in **d**. Quantifications are shown in **e**. Scale bars = 20 μm. **f** Average volume of conidia from the indicated strains recovered from oatmeal agar after 7 days. The data from 3 separate plates is given. Error bars are represented as the standard error from three replicates. Significant differences based on t-tests (*p* < 0.05) are marked with asterisks

In order to identify the mutation responsible for the phenotype of *vir-88*, we initially analyzed a T-DNA insertion site found in the original mutant screen which suggested the presence of two linked T-DNA insertions on supercontig5277 [26] close to CH063_12012 encoding a homolog to yeast Mob2 [25, 27]. By PCR analyses using a series of primers specific to that locus, both regions flanking the insertion could be isolated and were sequenced (Additional file 3: Text S1). The T-DNA insertion site was located upstream of the ATG in gene CH063_12012 and was accompanied by a small genomic deletion of 11 bp. Two T-DNA copies were inserted in a head to head fashion (Fig. 2a). Attempts to amplify the junction between the two T-DNA fragments by PCR were not successful, presumably due to the inverted arrangement of the sequences (not shown). The presence of two T-DNAs at this genomic context is consistent with the results of previous Southern Blot analysis probed with a hygromycin resistance DNA fragment [25]. CH063_12012 encodes a predicted 333 aa protein closely related to yeast Mob2. Because the T-DNA insertion was close to the annotated start codon and as the annotated N-terminus of CH063_12012 did not match the N-terminus of the yeast protein, it was important to verify the annotated gene model. In order to identify the correct coding region and complete transcript, we performed 5’- and 3’-RACE PCR (rapid amplification of cDNA ends) using total wild-type RNA as template (Additional file 3: Text S2). As shown in Fig. 2a, the resulting revised gene model for the *ChMOB2* gene consists of two 49 nt introns in the coding region, a 5’-UTR of 164 nt, a 3’-UTR of 216 nt and 834 nt of coding sequence. Accordingly, the T-DNA insertions of *vir-88* are located 161 bp upstream of the *ChMOB2* coding sequence. Interestingly, the putative *ChMOB2* transcription start at -164, as determined by 5’-RACE PCR, is located in the small genomic sequence which was replaced by T-DNA sequence in the genome of *vir-88*. Since the insertion is positioned upstream of *ChMOB2* and deletes the putative transcription start, we analyzed *ChMOB2* mRNA accumulation in RNA samples from *vir-88* and wild-type *C. higginsianum* by qualitative and quantitative PCR (Fig. 2b, c). In wild-type samples, *ChMOB2* RNA was detected in conidia, in appressoria formed on artificial surfaces, in mycelium and in infected *A. thaliana* leaves. RNA levels reached 20% relative to the housekeeping gene *ChTUBULIN-α* (CH063_01222) in appressoria and 55% in RNA from *A. thaliana* leaves 4 days after infection with wild-type *C. higginsianum. ChMOB2* was expressed strongly during appressoria formation as early as 4 h after inoculation (Fig. 2c). In contrast, *ChMOB2* transcript levels were reduced more than ten-fold in all *vir-88* samples (Fig. 2b, c). *ChMOB2* expression in *vir-88* infected *A. thaliana* could not be analyzed because virulence of this strain was too limited.

In order to verify that the insertion upstream of *ChMOB2* and the resulting strong reduction of *ChMOB2* expression is responsible for the phenotype of the *vir-88* mutant, we complemented *vir-88* both with a plasmid (pMOB2, pCK3110) harboring the *ChMOB2* wild-type allele from -1374 to +1797 and with a translational *ChMOB2*-GFP fusion under control of the strong and constitutive elongation factor 1 alpha promoter (pTef-*MOB2*-GFP, pCK4129). After reintroduction of wild-type *ChMOB2* by ATMT with pMOB2, virulence was fully restored (Fig. 2d, e). When conidia were recovered from oatmeal plates, their cell titers (not shown) and average cell size were restored to nearly wild-type levels (Fig. 2f). Transformation with pTef1-*MOB2*-GFP fully rescued the conidia cell size phenotype (Fig. 2f), while conidiation was restored to 80% of wild-type (not shown), similar to strains complemented with the wild-type allele. In the pTef1-*MOB2*-GFP transformants, virulence appeared to be even increased relative to the wild-type (Fig. 2d, e) possibly due to overexpression of *ChMOB2*. In all cases, empty vector controls (“EV” and “pTef-GFP”) did not affect the *vir-88* phenotypes. Since all observed phenotypes of *vir-88* can be complemented by reintroduction of *ChMOB2*, the phenotype of this mutant must be linked to the identified insertion. In summary, *vir-88* encodes a hypomorphic *ChMOB2* allele expressing less than 10% mRNA, which was generated by insertion of T-DNA in the 5’-UTR separating the coding region from its promoter.

### The *C. higginsianum* genome encodes three members of the Mob1/phocein protein family

**Fig. 3:**
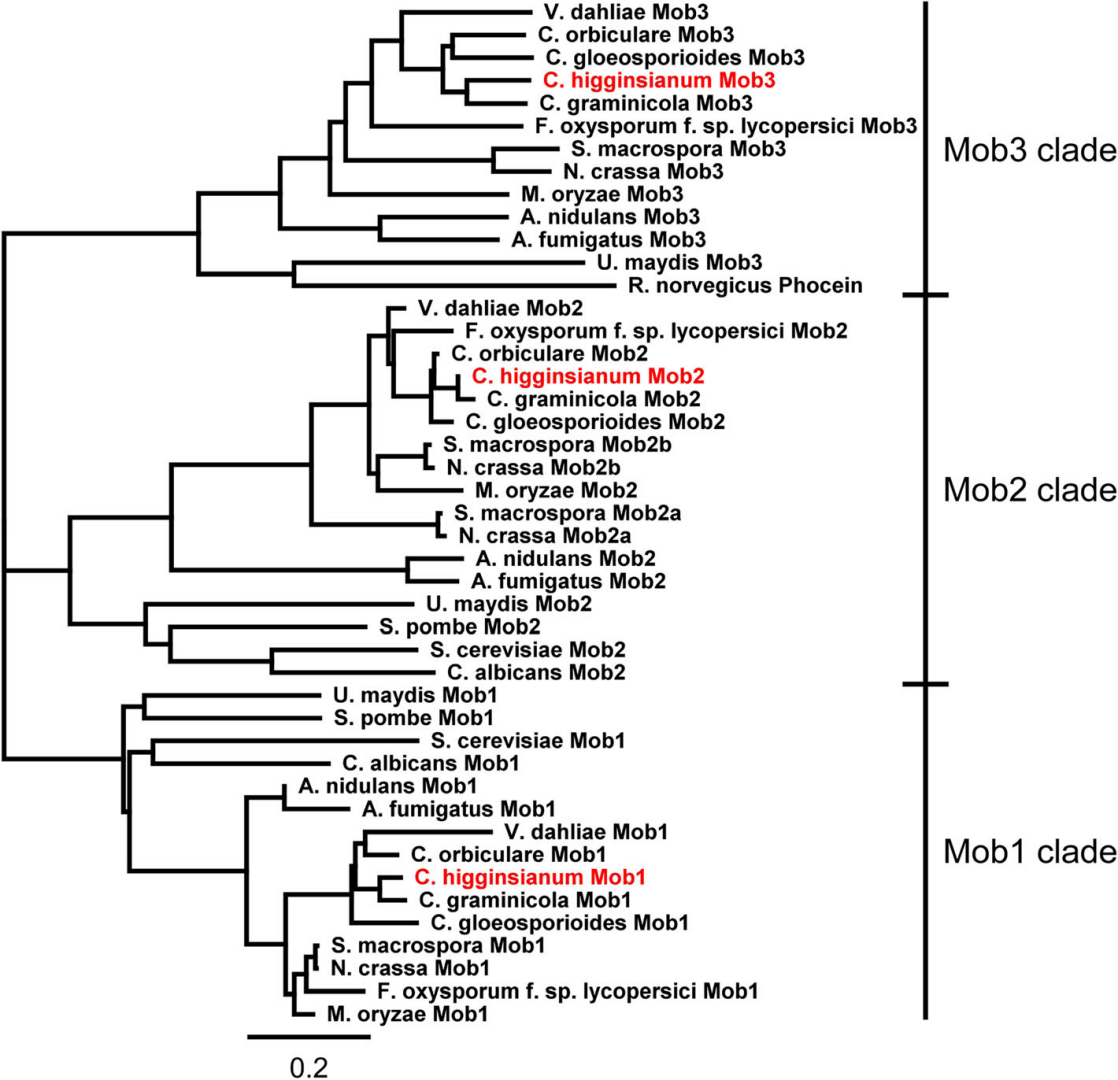
The genome of *C. higginsianum* encodes three Mob-like proteins. Unrooted phylogenetic tree of Mob proteins from *C. higginsianum* (marked in red) and other fungi. The tree was generated with the Geneious Tree builder plugin using a Blosum55 cost matrix and the Jukes-Cantor tree building method. The founding member of the Mob family (Phocein) from rat was included as a reference. Branch lengths correspond to substitutions per site. Scale bar = 0.2 substitutions per site

Mob2 belongs to the Mob1/phocein protein family, which is widely conserved among eukaryotes and many of its members have been shown to bind and activate NDR kinases [11, 15, 16, 22]. In contrast to metazoans, which contain up to six different Mob proteins [11], fungal genomes contain between two and four Mob coding genes. The resulting proteins can be grouped into three clades (Fig. 3): The first contains proteins with high similarity to yeast Mob1, which binds the Dbf2 kinase [13], while the second clade clusters together with yeast Mob2, which binds to Cbk1 [16]. The third clade contains phocein, the founding member of the Mob1/phocein family from *Rattus norvegicus* [24], and proteins from filamentous fungi like *S. macrospora* or *N. crassa* Mob3, which have been reported to be essential for development of reproductive protoperithecia and for hyphal fusion [22, 23]. All filamentous fungi analyzed in Fig. 3 contain one protein from each clade, except *S. macrospora* and *N. crassa*, which both harbor two *MOB2*-like genes. In *N. crassa*, Mob2a and Mob2b have partly overlapping functions and were both shown to interact with the Cbk1 like kinase Cot1 [22]. As *C. higginsianum* contains one protein from each clade, we assume that each Mob protein has functions similar to the respective homologs from its clade.

### *ChMOB2* and *ChCBK1* may be essential genes in *C. higginsianum*

In order to obtain *ΔChmob2* null mutants, we attempted to perform targeted gene knockout of *ChMOB2* by transformation of the non-homologous end-joining defective mutant *ΔChku80* [25] with the *ChMOB2* deletion plasmid pCK3712. This plasmid harbors the hygromycin resistance cassette (hph) flanked by *ChMOB2* upstream (-1015 to -198) and downstream sequence (+704 to +1513) on the T-DNA. In the *ΔChku80* background >90% of all transforming DNA should integrate by homologous recombination [25]. In three independent experiments, however, we recovered only transformants that harbored ectopically integrated DNA (a total of six). In contrast, transformations of the NHEJ proficient wild-type strain yielded >160 transformants. We therefore expect that *ChMOB2* may be an essential gene in *C. higginsianum*, at least in the *ΔChku80* background*.* Interestingly, *MOB2* can be inactivated in *S. cerevisiae* and in *N. crassa* [22, 27]. As described for *S. cerevisiae*, Mob family proteins often act as co-activators for NDR/LATS kinases. In particular, Mob2 binds to Cbk1 kinase in yeast [16]. We assumed that ChMob2 has a similar function in *C. higginsianum* and may act through activation of ChCbk1. We attempted to delete *ChCBK1* (CH063_12968) in the *ΔChku80* background. In three independent experiments this failed similar to *ChMOB2* (not shown). It is therefore likely that both *ChMOB2* and *ChCBK1* have essential functions in *C. higginsianum*. In addition, attempts to generate knock-down mutants of these genes by expression of antisense-RNAs did not lead to transformants with significantly reduced levels of the respective transcripts (Additional file 4: Figure S3).

### ChMob2 co-localizes with the potential kinase ChCbk1 in appressoria

**Fig. 4:**
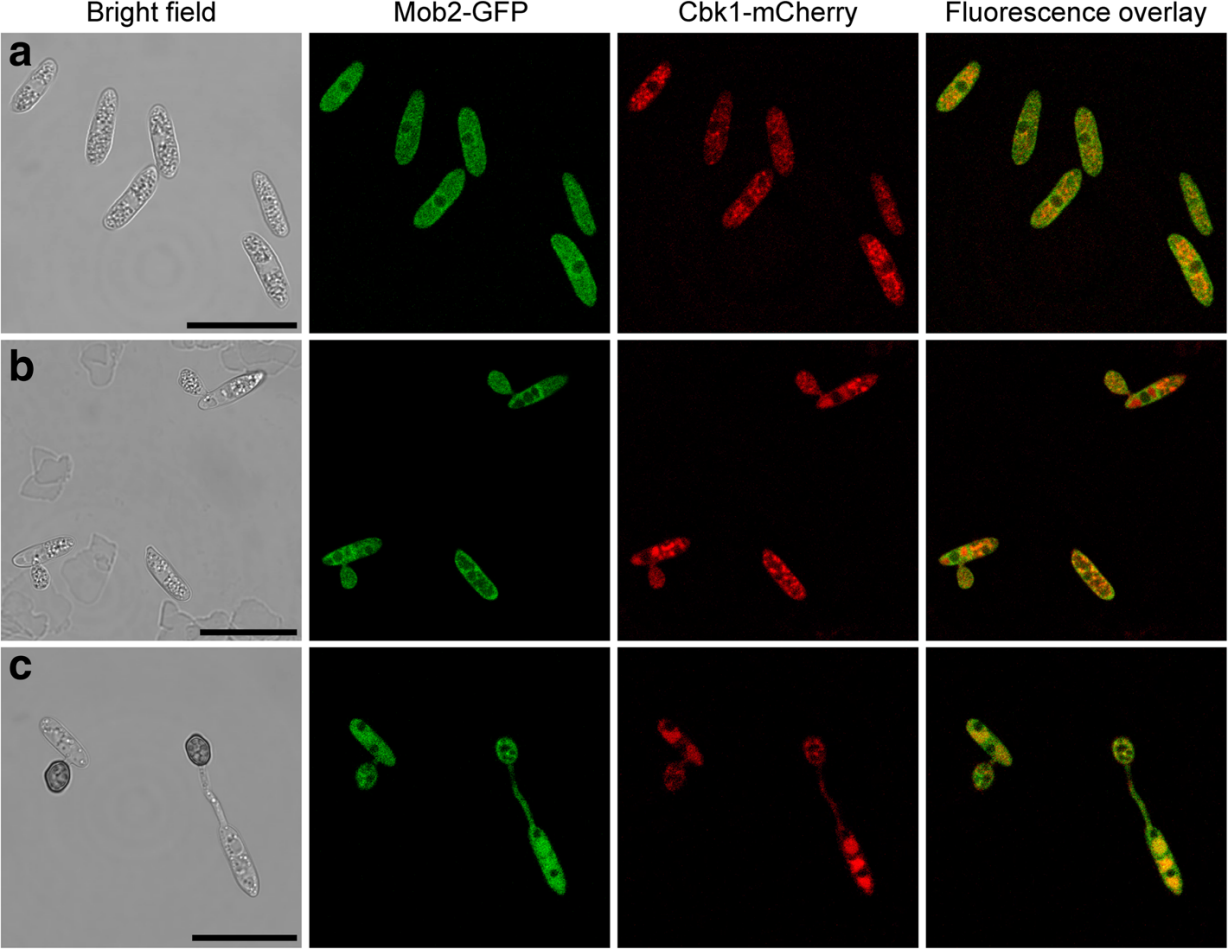
ChMob2 co-localizes with ChCbk1 in the cytoplasm. Conidia of Δ*Chcbk1*:: *CBK1*-mCherry Δ*Chmob2*:: *MOB2*-GFP (CY6720) were rinsed off oatmeal plates and analyzed by CLSM during in vitro appressoria formation. **a** Conidia, **b** in vitro AP at 8 hpi, **c** in vitro AP at 18 hpi. Bright field, GFP and mCherry channels are shown from left to right. The rightmost picture shows an overlay of GFP and mCherry channels. The intensities between panels are not directly comparable, because different settings were used to optimally visualize protein localization. Scale bars = 20 μm

In order to further examine the function of *C. higginsianum* Mob2, we investigated the subcellular localization of ChMob2 and its potential binding partner ChCbk1. We constructed a strain (CY6720: *ΔChku80 ΔChcbk1*:: *CBK1*-mCherry *ΔChmob2*:: *MOB2*-GFP) where the genomic copy of *ChCBK1* was replaced by a *CBK1*-mCherry fusion and where *ChMOB2* was simultaneously replaced by *MOB2*-GFP. The resulting strain had no significant defects in either virulence, conidiation or vegetative growth except for producing slightly smaller conidia (-12% compared to the parental strain *ΔChku80*, Additional file 5: Figure S4). Since both *ChMOB2* and *ChCBK1* may be essential in *C. higginsianum*, reduced ChMob2 or ChCbk1 function should have obvious effects. We therefore concluded that both fusion proteins were functional. In conidia isolated from OMA plates (Fig. 4a), both fusion proteins were more or less evenly distributed over the whole cell except for the nucleus, which appeared to be partly excluded. The two proteins co-localized to some extent. Early upon in vitro appressoria formation, when appressoria were unmelanized (Fig. 4b), both Cbk1-mCherry and Mob2-GFP were completely overlapping. In the germinated conidia attached to these appressoria, however, the fusion proteins seemed to be localized to different compartments. In mature appressoria and their corresponding germinated conidia (Fig. 4c), both fusion proteins were again co-localized all over the cells except in nuclei, where both were absent. In axenic cultures, co-localization could not be addressed because the Cbk1-mCherry fusion was barely detectable (not shown). *In planta*, we could not detect either of the two fusion proteins, presumably because of the high background fluorescence in green leaves (not shown). Taken together, Mob2-GFP and Cbk1-mCherry show the same subcellular localization during appressoria formation.

### Mob2-GFP physically interacts with Cbk1-HA

**Fig. 5:**
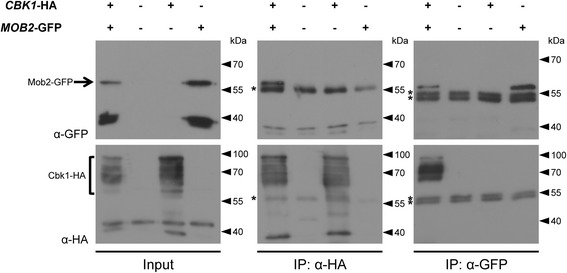
ChMob2 interacts with ChCbk1. Whole cell extracts from strains expressing different combinations of ChMob2-GFP and ChCbk1-HA were used to analyze the interaction of ChMob2 and ChCbk1 by immunoprecipitation. Strains used from left to right: Δ*Chcbk1*:: *CBK1*-HA pTef1a-*MOB2*-GFP (CY6681), the parental strain Δ*Chku80* (CY6021), Δ*Chcbk1*:: *CBK1*-HA (CY6678) and pTef1a-*MOB2*-GFP (CY6543). Western blots of whole cell protein extracts (input), immunoprecipitates with anti-HA antibody coated beads (IP: α-HA) and immunoprecipitates with anti-GFP antibody coated beads (IP: α-GFP) were decorated either with anti-GFP (upper panels) or with anti-HA (lower panels) primary antibodies. The band corresponding to Mob2-GFP is marked with an arrow. The multiple bands corresponding to Cbk1-HA are marked with a bracket. Asterisks mark bands corresponding to primary antibody which are present in the IP and therefore are recognized by the secondary antibody

ChMob2 and ChCbk1 can occupy the same subcellular compartment. Consequently, they might physically interact with each other during certain times in the cell cycle. In order to test their possible interaction, we performed co-immunoprecipitation of ChMob2 and ChCbk1. We constructed a strain (CY6681) where *ChCBK1* was fused to a 6xHA tag at its own genetic locus. In addition, this strain ectopically expressed *MOB2*-GFP under control of the Tef1a promoter. Both proteins could be detected in axenic mycelium after western blotting with anti-GFP and anti-HA antibodies (Fig 5, input). Mob2-GFP was detected at about 60 kDa (Fig. 5, input). Cbk1-HA produced several bands between 60 and 95 kDa, possibly because of different phosphorylation states of the protein or due to protein degradation (Fig. 5, Input). We used protein extracts from mycelium to investigate the interaction of ChCbk1 with ChMob2. First, Cbk1-HA was immunoprecipitated with anti-HA antibodies coupled to magnetic beads (Fig. 5, IP: α-HA). The successful pull down of Cbk1-HA is shown in the western blot decorated with anti-HA antibodies (Fig. 5 IP: α-HA, lower panel). The presence of Mob2-GFP in these immunoprecipitates was determined by decorating the western blots with anti-GFP antibodies (Fig. 5, IP: α-HA, upper panel). In extracts containing both fusion proteins (Fig. 5, IP: α-HA, lane 1), Mob2-GFP was efficiently co-immunoprecipitated with Cbk1-HA (Fig. 5, IP: α-HA, upper panel, lane 1), but not from control extracts lacking Cbk1-HA (Fig. 5, IP: α-HA, upper panel, lane 4). Similarly, when Mob2-GFP was pulled down with anti-GFP antibody coated beads, Cbk1-HA was co-precipitated (Fig. 5, IP: α-GFP, lower panel, lane 1). Some Cbk1-HA bands were missing from the anti-GFP IP, suggesting that at least some of the ChCbk1 forms might not bind to ChMob2. The successful co-precipitation of ChMob2 and ChCbk1 indicates that the two proteins can interact in axenic *C. higginsianum* cells. While technically not testable, it is likely that this interaction also occurs during infection.

Next, we tried to verify this interaction by mass spectrometry using Cbk1-HA expressing cell extracts without overexpressing Mob2-GFP. This approach could potentially also identify downstream targets of ChCbk1-phosphorylation and could show whether or not ChMob2 is the sole activator of ChCbk1. We pulled down Cbk1-HA from extracts of axenic mycelium of CY6678 (*ΔChcbk1*:: *CBK1*-HA) and from extracts of the parental strain as control in two biological replicates using anti-HA antibody-coated magnetic beads. The eluates were analyzed by Nano LC-MS/MS and quantified label-free by integrating peak areas (Additional file 6: Table S1). ChMob2 was only identified in the *CBK1*-HA IPs but not in samples from the untagged parental strain, further verifying the interaction of these two proteins in *C. higginsianum*. The other peptides identified were probably contaminants as they consisted mainly of highly expressed enzymes from the citrate cycle, from glycolysis or proteins associated with ribosomes. The lack of potential ChCbk1 targets in the pull-downs is likely due to transient interactions of the kinase with its substrates. Alternatively, the important proteins may not be present or not targeted by ChCbk1 in samples of in vitro mycelium.

### Two potential downstream targets of the ChMob2/ChCbk1 complex are required for full pathogenicity

**Fig. 6:**
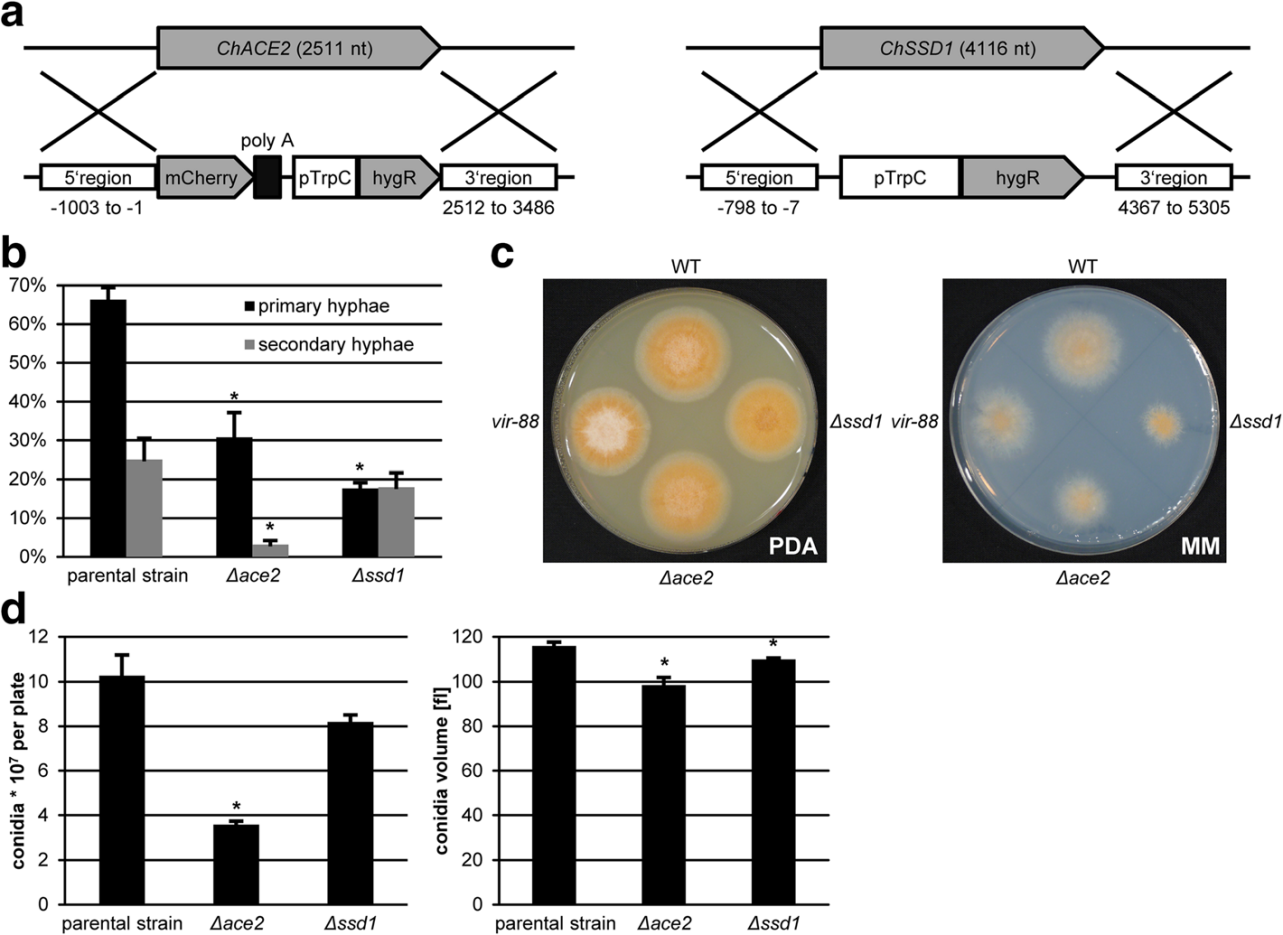
*ChACE2* and *ChSSD1* are required for full virulence of *C. higginsianum*. **a** Schematic representation of the genomic loci of *ChACE2* and *ChSSD1* (top) and the knockout cassettes of the respective deletion plasmids (bottom). The position of 5’ and 3’ flanking regions are given relative to the respective start codon. **b** Spray infection of *A. thaliana* at 3 dpi*.* Primary hyphae (relative to appressoria) and secondary hyphae (relative to primary hyphae) produced by the indicated strains. **c** Vegetative growth of the indicated strains on potato dextrose agar (PDA) and Czapek-Dox agar minimal medium (MM). **d** Average number of conidia (left) and cell volume (right) for the indicated strains recovered from oatmeal agar after 7 days. The data from 3 separate plates is shown. The strains used in **b**-**d** were wild-type (CY5535), *vir-88*, Δ*Chace2* (CY6353), Δ*Chssd1* (CY6649) and the Δ*Chku80* parental strain of the last two (CY6021)*.* Error bars are represented as the standard error from three replicates. Significant differences based on t-tests (*p* < 0.05) are marked with asterisks

Two well described Cbk1 targets in yeast are the transcription factor Ace2 and the mRNA-binding protein Ssd1 [16, 18]. We therefore tested whether or not their orthologs from *C. higginsianum* are involved in pathogenicity. We identified possible orthologs in the *C. higginsianum* genome [26]. The closest homolog to ScAce2 was CH063_01293 with 24.1% identity. After comparison with the sequence of *S. cerevisiae* Ace2 and the more closely related AfAce2 of *A. fumigatus* [28], we concluded that the automatic annotation of CH063_01293 was not complete and extended the N-terminus by 271 amino acids resulting in a revised *C. higginsianum ChACE2* gene model encoding for 815 amino acids. This extension could be verified by sequencing *ChACE2* cDNA (not shown). The revised protein has 19.5% sequence identity to ScAce2 and 31.9% to AfAce2 over the full sequence. The region containing the C2H2 zinc finger DNA binding domain showed 65.8% sequence identity to AfAce2. The potential *C. higginsianum* Ssd1 (Accession BAI59007 [29]) shows 34.5%, 77.9% and 93.2% sequence identity to the potential orthologs from *Saccharomyces cerevisiae* (Accession NP_010579), *Magnaporthe grisea* (Accession XP_003715039) and *Colletotrichum lagenarium* (Accession BAE66713), respectively. Although both ChSsd1 and ChAce2 show less than 40% identity to their respective yeast homologs, they are clearly the best match in reciprocal BLAST searches (not shown). We generated *ΔChace2* (CY6353) and *ΔChssd1* (CY6649) deletion strains in the *ΔChku80* background and examined their phenotype (Fig. 6). Consistent with a role of ChAce2 and ChSsd1 downstream of the ChMob2/ChCbk1 complex, both mutants exhibited significantly reduced virulence (Fig. 6b). Furthermore, *ΔChace2* and *ΔChssd1* strains showed decreased vegetative fitness, as their colonies on minimal medium were smaller by about 35% and 50% than wild-type, respectively (Fig. 6c). None of the mutants showed significantly altered sensitivity to osmotic stress, heat, H_2_O_2_, or cell wall stress (Additional file 2: Figure S2). Interestingly, conidial cell size and especially the conidial titer recovered from OMA plates were reduced in the *ΔChace2* mutant (Fig. 6d) similar to the phenotype observed in *vir-88*. The phenotype of *ΔChace2* could be rescued by reintroduction of wild-type *ChACE2* into the *ΔChku80* locus of this strain (Additional file 7: Figure S5), further confirming the involvement of ChAce2 in virulence and conidiation. The decrease in fitness of the *ΔChace2* and *ΔChssd1* mutants could contribute to their virulence phenotype. The conidiation phenotype of the *ΔChace2* mutant, however, is likely to be a specific phenotype, as slow growing mutants do not a priori produce less conidia (Fig. 6d). It was not possible to generate a *ΔChace2 ΔChssd1* double mutant (not shown).

In order to analyze if ChAce2 is regulated by the ChMob2/ChCbk1 complex in *C. higginsianum* as in *S. cerevisiae* [16], we analyzed the mRNA amount of potential orthologs of the ScAce2 targets *CTS1* and *SCW11* [19, 30] and of the *S. pombe* Ace2 target *MID2* [31] in samples from *vir-88* and *ΔChace2*. We expected that if ChAce2 activity were regulated by ChMob2, the transcript amount of ChAce2 targets might be different in *vir-88*, which expresses only little *ChMOB2* (Fig. 2b), and in *ΔChace2* mutants, which lacks *ChACE2*. However, using both semi-quantitative and quantitative RT-PCR we could not detect significant *ChCTS1*, *ChSCW11* or *ChMID2* transcript changes in appressoria samples in comparison to the wild-type (Additional file 8: Figure S6; see also Fig. 2c). The mRNA level of *ChACE2* was not significantly different between *vir-88* and the wild-type (Additional file 8: Figure S6). This could indicate that similar to the situation in yeast [16], ChAce2 is not regulated on the level of mRNA accumulation but by ChCbk1-dependent phosphorylation. Furthermore, *ΔChcts1* strains (CY7110 and CY7111), which lack one of the genes predicted to encode chitinase, did not show any defects in virulence, conidiation or cell separation (Additional file 8: Figure S7). A cell separation phenotype was observed in yeast *Δcts1* mutants [32]. This may indicate that Ace2 regulates different targets in *C. higginsianum* than it does in yeasts. In summary, we were not able to identify downstream targets of ChMob2/ChCbk1 in *C. higginsianum*, but deletion of two Cbk1 targets known from other systems resulted in pathogenicity and conidiation phenotypes resembling those of *vir-88*.

### The roles of ChMob3 and ChMob1 in *C. higginsianum*

While *MOB1* and *MOB2* seem to be present in all fungal genomes, *MOB3* is only found in filamentous fungi and higher eukaryotes [22]. The mammalian ortholog of Mob3 (Phocein) was identified in rat dendritic cells and has been shown to bind to striatin, a calmodulin binding protein [24]. In fungi, *MOB3* has been reported to be essential for sexual development and for hyphal fusion in *N. crassa* and *S. macrospora* [22, 23]. Although *C. higginsianum* has not been described to perform a sexual cycle, a potential ortholog of this gene is encoded in the *C. higginsianum* genome. We therefore investigated the potential roles of ChMob3 in *C. higginsianum* by targeted gene knockout of *ChMOB3 (CH063_02262)*. Except for a very mild reduction in colony size on both PDA and minimal medium, we did not observe significant changes either in virulence or conidiation of the resulting *ΔChmob3* mutants (Additional file 9: Figure S8). The expression pattern of *ChMOB3* did not give any further hint regarding its physiological function, as it was expressed at low, constitutive levels under all tested conditions (not shown). We cannot exclude that ChMob3 has a specific function under different, specific conditions.

**Fig 7:**
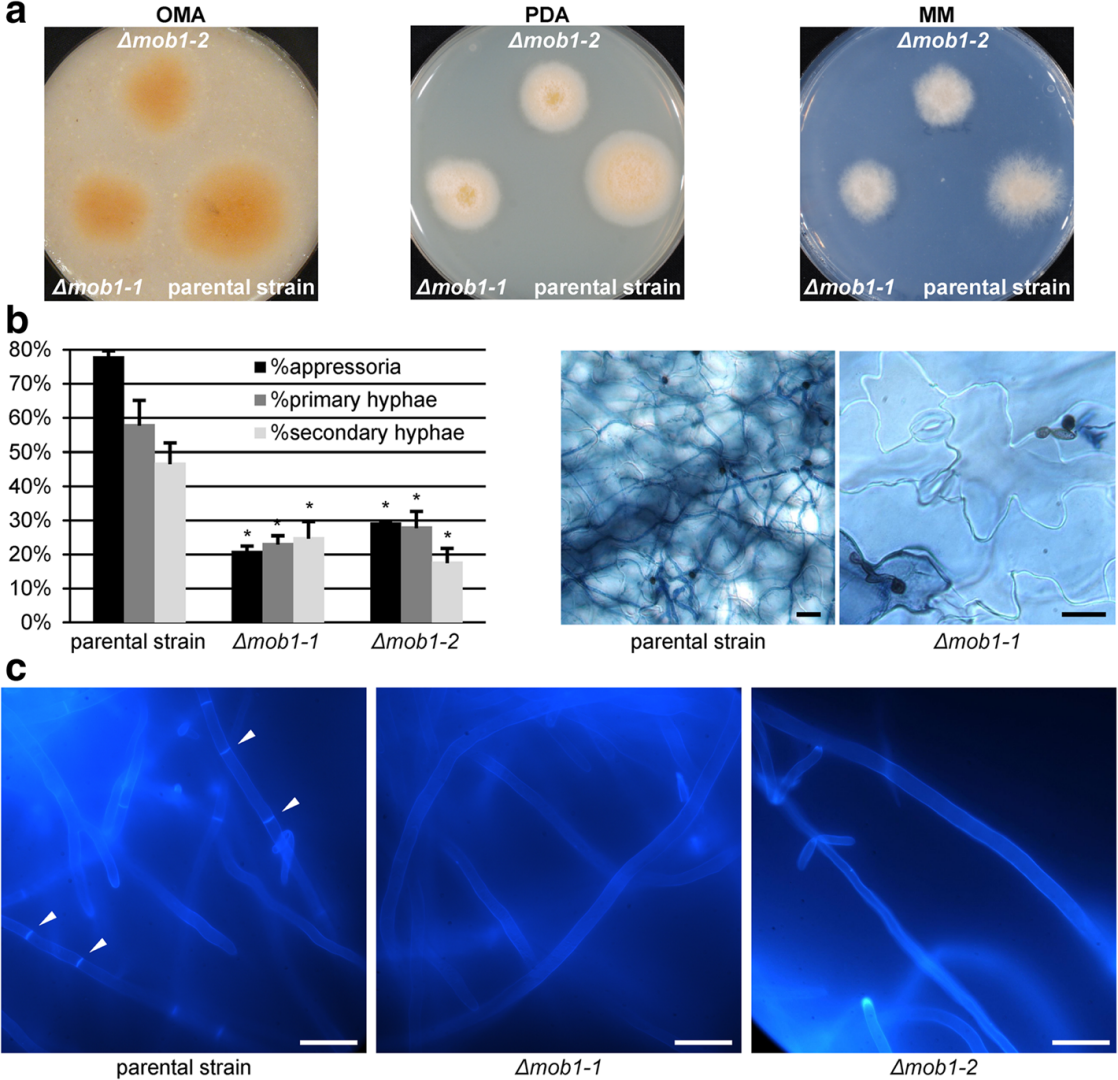
*ChMOB1* is required for septum formation and cell division. Two independent Δ*Chmob1* transformants (CY7242 and CY7243) and the parental strain Δ*Chku80* (CY6021) were tested for vegetative growth, virulence and the ability to produce septae. **a** Vegetative growth on oatmeal agar (OMA), potato dextrose agar (PDA) and Czapek-Dox minimal medium (MM). **b** *A. thaliana* 4 days after droplet inoculation. Representative images of trypan blue stained leaves are shown on the right. The quantification of appressoria (relative to total conidia titer used for infection), primary hyphae (relative to appressoria) and secondary hyphae (relative to primary hyphae) of this infection is shown on the left. The data of 3 plants per strain with 2 leaves per plant with at least 100 counted appressoria per leaf is given. Error bars are represented as the standard error from three replicates. Significant differences based on t-tests (*p* < 0.05) are marked with asterisks. **c** Axenic mycelium stained with calcofluor white. Septae are marked with white arrows. Scale bars = 20 μm

In yeasts and *N. crassa*, Mob1 and Mob2 have distinct, but similar functions, namely the binding and activation of a NDR/LATS-kinase [13, 15, 16, 22]. In *S. cerevisiae*, Mob2 binds to Cbk1 in the RAM pathway and Mob1 binds to Dbf2 in the mitotic exit network. Although binding of Mob1 or Mob2 to their respective kinases is thought to be specific [15, 22], there may be crosstalk between MEN and RAM networks in *S. cerevisiae* [20, 33]. We therefore investigated if ChMob1 and ChMob2 have overlapping functions in *C. higginsianum* by genetically inactivating *ChMOB1* (CH063_02209). Transformation of *ΔChku80* with a *ChMOB1* deletion plasmid resulted in mutants with a strong growth phenotype on all tested media (Fig. 7a). In addition, these mutants showed strong sensitivity to the cell wall stress inducing and chitin binding dye congo red (Additional file 2: Figure S2), indicating a defect in chitin turnover in the cell wall [34]. Furthermore, *ΔChmob1* mutants hardly produced any conidia on oatmeal medium (not shown). The few conidia that could be recovered were smaller by about 50% (not shown) and were significantly reduced in formation of appressoria, primary hyphae and secondary hyphae after droplet inoculations (Fig. 7b). In contrast to wild-type conidia which form a septum during germination, *ΔChmob1* conidia were never septated (Additional file 9: Figure S9). Furthermore, *ΔChmob1* conidia were often connected to 1 or 2 other conidia, possibly because they did not undergo proper cell separation during conidiation (Additional file 9: Figure S9). Hyphae from axenic mycelium of *ΔChmob1* strains also hardly produced any septae at all, in contrast to the parental strain (Fig. 7c). The strong septation phenotype of *ΔChmob1* strains is similar to mutants in *S. cerevisiae* and *S. pombe*, where Mob1 is required for completion of mitosis [27], initiation of cytokinesis and septation [35].

In summary, *C. higginsianum* contains two additional *MOB* genes. Consistent with a function of Mob3 during sexual development in other systems [22, 23], deletion of *ChMOB3* did not lead to any significant phenotype in *C. higginsianum* which is lacking a sexual cycle. In contrast, knockout of *ChMOB1* leads strong defects in appressoria formation, vegetative growth, septation, conidiation and infection.

## Discussion

Screening for genes involved in pathogenicity though aimed at identification of genes with functions exclusively required for certain aspects of the infection process, will always also identify genes with vegetative functions at many stages of their life cycle. ATPases [25, 36], intracellular transport [37], nutrition [38] and morphogenesis [39] are notable examples. Appressorial pathogens like *Colletotrichum higginsianum* undergo switches in polarity during infection of plant cells. In particular, the directed formation of a penetration peg underneath the appressorium is essential for infection. It therefore is not too surprising to identify polarity determinants like Mob2 in a screen for pathogenicity genes.

We identified a weak allele of *ChMOB2* in the *vir-88* mutant that in most cases produces appressoria with morphologic defects like elongation or secondary outgrowths. These appressoria are not able to penetrate and to initiate the infection process. Since a fraction of the *vir-88* appressoria looked normal and appeared functional, the mutant shows residual weak symptoms in some spots on the leaf, resulting in a mutant with reduced pathogenicity. In addition, *vir-88* produces less and smaller conidia. Although *vir-88* expresses less than 10% of ChMob2 mRNA relative to the wild-type, it is not a null mutant. Since the T-DNA insertion in the genome of *vir-88* is located just in front of the *ChMOB2* reading frame, it is likely that residual ChMob2 activity may be present, at least in some cells. The phenotype of a true null mutant could not be analyzed because all attempts to generate a deletion allele by homologous recombination failed. The likely explanation, therefore, is that *ChMOB2* is an essential gene at least in the *ΔChku80* background used for all knockouts. A low expression, which may vary from cell to cell, could also be the reason why not all mutant cells behave identical, resulting in incomplete penetrance of the underlying genetic defect. While this makes interpretation of the phenotype more difficult, the fortuitous isolation of a weak allele allowed us to characterize the functions of ChMob2. The defect of most *vir-88* appressoria to penetrate the host tissue correlated with morphological defects. In agreement with a possible essential function for ChMob2, its binding partner, the NDR/LATS kinase ChCbk1, could also not be knocked out. While a failure to generate knockout mutants is no evidence for an essential function, it resembles the situation in yeast and *S. pombe*, where Mob2 and Cbk1 have been found to be essential. [15, 40]. Interestingly, the lethality of RAM mutations in yeast is suppressed by loss of Ssd1 [41–43], showing that the consequences of RAM mutations may be strain specific. In most systems, *mob2* and *cbk1* mutations are indeed associated with pleiotropic phenotypes and morphological defects. In the opportunistic human pathogen *C. albicans*, all mutants in the RAM pathway including CaMob2 and CaCbk1 are viable but show hyperpolarization [44]. In the basidiomycetes *C. neoformans* and *U. maydis*, mutants of the Cbk1 ortholog Ukc1, Mob2 and all other associated proteins thought to be involved in the same pathway lead to hyperpolarized growth and decreased virulence [45, 46]. In *A. nidulans,* mutants in Cbk1 (AnCotA) and Mob2 orthologs (AnMobB) show growth defects, strongly reduced ability to form conidia, altered conidia cell size and increased number of nuclei in spores [47, 48]. In *N. crassa*, which is more closely related to *C. higginsianum,* the *cot-1 (ts)* mutant and the *mob2a mob2b* double mutant show increased rate of branching in axenic mycelium, reduced growth rate and reduced conidiation [21, 22]. Most phenotypes observed in our study for the *Chmob2* mutant *vir-88* are consistent with functions reported for Mob2 and Cbk1 in filamentous fungi, including a function in conidiation. Reduced cell size of conidia was an obvious phenotype of *vir-88*. Altered conidial cell size may be the consequence of altered cell division or septation in conidial precursor cells [49]. Altered conidial cell size was also observed in *N. crassa cot1* mutants. They were, however, 4 times larger than wild-type cells, rather than smaller as in the case of *C. higginsianum* [48]. We also observed about 50% reduction in conidiation ability in the *vir-88* mutant. Considering that *vir-88* is not a null allele of *ChMOB2*, this phenotype is reminiscent of *A. nidulans* and *N. crassa mob2* mutants which largely fail to form conidia [22, 47]. Conidiation is critically dependent on septation, which in turn is also dependent on the Mob1/DBF2-dependent MEN (Mitotic Exit Network) or SIN (Septation Initiation Network) signalling pathways in many fungi [13, 22, 50–52]. In fact, *N. crassa* and *A. nidulans* *Δmob1* [22, 50] show sporulation defects similar to the *C. higginsianum ΔChmob1* mutants described in this study. It is possible that the Mob1/Dbf2 and Mob2/Cbk1 complexes have overlapping functions or that they control each other. The latter may be the case in related systems. In *N. crassa,* Mob1 appears to affect Cot1 activity more than its direct interactor and co-activator Mob2b [22]. In *S. pombe*, SpSid1 (= Dbf2) controls SpOrb6 (= Cbk1) activity [53], while Mob1 and Cdc14 activity is required for proper Mob2 and Ace2 localization in yeast [16]. For comparison, a simplified model of the potential interplay between MEN and RAM of *S. cerevisiae* is shown in Additional file 10: Figure S10.

In addition to a strong conidiation phenotype, *ΔChmob1* mutants exhibited attenuated ability to form appressoria, primary and secondary hyphae upon infection and showed a severe septation defect. These phenotypes are similar to the corresponding mutants in *N. crassa* [22] and *A. nidulans* [50]. Mob1 is essential both in S. *cerevisiae* [27] and *S. pombe* [14], but cells harboring conditional alleles of *MOB1* either arrest in late mitosis or, in the case of *S. pombe*, do not septate and become multinucleate. In addition to nuclear division, *S. cerevisiae* Mob1 also seems to have a role during cytokinesis and cell separation [54]. Based on these similarities, it is likely that the function of Mob1 and its associated signalling pathway is conserved among ascomycetes.

What are the critical targets of the Mob2/Cbk1 pathway in filamentous fungi? Similar to the situation in yeast, it was found that deletion of the *N. crassa* homolog of *SSD1* (*GUL1*) can suppress defects of the *cot1 (ts)* allele [55, 56] suggesting that Ssd1 is an important component of the RAM pathway also in filamentous fungi. In *S. cerevisiae*, Ssd1 inactivation is thought to occur through Cbk1 phosphorylation [41–43]. Because Ssd1 may be a conserved Cbk1 target we analyzed the phenotype of a *ΔChssd1* mutant allele. We found that the lack of ChSsd1 leads to attenuated virulence in *C. higginsianum*. The effect on virulence was similar to *vir-88* and to *Δssd1* mutants in the plant pathogens *C. lagenarium* and *Magnaporthe grisea* [57] supporting the notion that Ssd1 is a conserved virulence associated target of the RAM pathway. In *C. lagenarium* [57], *Δssd1* mutants mostly failed to penetrate and intracellular hyphae were only detected very rarely. In *M. grisea* [57], *Δssd1* mutants were still able to produce intracellular primary hyphae on rice plants, but they were mostly restricted to dead host cells.

We also analyzed the effect of ChAce2 on pathogenicity in *C. higginsianum* because it may be a conserved target of the RAM pathway. Again, we saw a reduction of pathogenicity and, interestingly, a comparable effect on cell volume in conidia and reduced conidiation. However, we found no evidence for posttranslational regulation of ChAce2 by the ChMob2/ChCbk1 complex, as described for *S. cerevisiae* [12].

We observed that *C. higginsianum* Mob2 and Cbk1 localize to the cytoplasm and are excluded from nuclei in conidia and during in vitro appressoria formation. In baker’s yeast, however, Mob2 and Cbk1 proteins can be found at sites of polarized growth like the cortex of the growing bud and the mating projection or in the daughter cell nucleus and the septum during cell separation, where they supposedly regulate cytokinesis genes through the transcription factor ScAce2 [16]. This difference in localization could indicate that Ace2 is not a conserved Mob2/Cbk1 target in filamentous fungi. In the absence of verified targets for the transcription factor ChAce2, it also remains unclear whether or not ChAce2 and yeast Ace2 have a common function. A protein homologous to ScAce2 was also analyzed in *A. fumigatus* [28], where knockout mutants had attenuated ability to produce conidia, abnormal cell wall architecture and were hypervirulent in a mouse model. This protein (AfAce2) is more closely related to ChAce2 (31.9% sequence identity) than to the yeast protein and, given their common conidiation phenotype, they may be orthologous gene products.

## Conclusion

This study showed that the Mob-family protein ChMob2 from the plant pathogen *Colletotrichum higginsianum* is involved in virulence on *Arabidopsis thaliana* and has a role in conidiation. ChMob2 forms a complex with the conserved ChCbk1 Kinase. The study further analyzed the functions of the other two Mob proteins encoded in *C. higginsianum* by targeted gene knockouts. *ChMOB1* is required for conidiation, cytokinesis and plant infection, while *ΔChmob3* mutants have no obvious phenotype in vegetative cells or during infection.

## Methods

### Strains and media

*E. coli*, *A. tumefaciens* and *C. higginsianum* strains were obtained, cultured and transformed as described [25] with the following modification: For selection of bialaphos-resistant *C. higginsianum* transformants, the transformation mixture of conidia and *Agrobacteria* was co-cultivated on Czapek Dox minimal medium plates containing 2.64 g/l (NH_4_)_2_SO_4_. For selection, the plates were supplemented with 10 – 20 μg/ml bialaphos and 100 μg/ml cefotaxime. *A. thaliana* Col-0 was grown and infected with *C. higginsianum* as described [25]. In vitro appressoria were induced on petri dishes as described [25]. For analysis of conidiation, *strains* were grown for 7 days on oatmeal plates and then stored at 4 °C. Conidia were rinsed off with sterile water, washed once by centrifugation for 5 min at 3000 rpm and measured by cell counting. Three wells of an oatmeal agar 12-well petri dish were inoculated with 100 μl of conidia suspension containing 1000 conidia. After incubation for 7 to 8 days, each well was thoroughly harvested three times with 500 μl MQ. The three aliquots were pooled and measured by cell counting. This experiment was repeated at least once for every strain. All *C. higginsianum* strains used in this study are listed in Additional file 11: Text S3.

### Standard techniques

DNA manipulations, PCR reactions, Southern blotting and plasmid DNA isolations followed standard protocols as described [25, 58]. Extraction of RNA, RT-PCR and qRT-PCR were performed as described [25]. Oligonucleotide sequences and plasmid constructions are listed in Additional file 11: Text S3.

### Microscopy and histochemical staining

Histochemical samples or samples containing fluorescent reporter proteins were stained and analyzed by microscopy as described [25] with the following modifications. Confocal GFP fluorescence was detected between 498 and 547 nm and mCherry fluorescence between 570 and 637 nm. Quantification of trypan blue stained fungal infection structures after spray infection was performed with 3 infected plants per strain at 3 days post infection. Two leaves were analyzed per plant. At least 400 appressoria were counted per leaf. Septae were stained with calcofluor white (100 fold dilution with PBS buffer of a 10 mg/ml stock solution in DMSO) by addition of the working solution to the specimen and rinsing 2 to 3 times with water. Fluorescence was observed using the DAPI filter.

### Sequence analyses and accessions

*C. higginsianum* DNA sequences [59] were obtained from the *Colletotrichum* Sequencing Project, Broad Institute of Harvard and MIT [26] and from the EnsemblFungi Server [60]. Where indicated, DNA sequences were obtained from the Max Planck Institute for Plant Breeding Research *Colletotrichum higginsianum* Database [61] and [62]. Sequence alignments and phylogenetic trees were performed with Geneious Alignment or ClustalW algorithms as implemented in the Geneious 5.5.6 software package (Biomatters Limited).

Genbank Accessions: *ChMOB1* (CCF39800), *ChMOB2* (KP261084), *ChMOB3* (CCF40036), *ChACE2* (BK009983), *ChSSD1* (AB508804). Additional accessions can be found in Additional file 11: Text S3.

### Rapid amplification of cDNA ends (RACE)

5’- and 3’- RACE PCR was performed as described in the SMART™ RACE cDNA Amplification Kit User Manual (Clontech) with the following modifications. 5’RACE PCR-ready cDNA was prepared using 1 μg of total RNA isolated from conidia, 1 μl of 5’CDS primer (12 μM, CK4502), 1 μl of SMART II A oligo (12 μM, CK3983) and 1 μl Powerscript reverse transcriptase (Clontech) in a total volume of 8 μl. 3’RACE PCR-ready cDNA was prepared identically except for 1 μl of 3’CDS primer (12 μM) instead of the 5’CDS primer and without SMART II A oligo. ChMOB2 5’ RACE PCR was performed using 2.5 μl of 5’RACE PCR-ready cDNA as template and 5 μl universal primer mix (UPM, 0.4 μM CK3951 and 2 μM CK3952) together with 1 μl CK4050 (10 μM). ChMOB2 3’RACE PCR was performed using 2.5 μl 3’RACE PCR-ready cDNA and 5 μl UPM together with 1 μl CK4048 (10 μM). These reactions were set up in a total volume of 50 μl and contained 1.25 U HotStart Taq (Peqlab), 5 μl reaction buffer S and 1 μl dNTPs (10 mM each) in addition to template and primers. The reaction products were diluted 50 fold in Tricine-EDTA buffer and used as templates for the nested ChMOB2 RACE PCR with the nested universal primer mix (NUPM) together with CK4049 (5’RACE PCR) or CK3971 (3’RACE PCR). The resulting ChMOB2 PCR products of approximately 250 bp (5’RACE PCR) and 1100 bp (3’RACE PCR) were subcloned into CloneJet (Thermo Fisher Scientific) and sequenced.

### Co-immunoprecipitation of *C. higginsianum* extracts

6 x 10^5^ conidia (washed once) were inoculated in 300 ml liquid Modified Mathur’s medium [63] and incubated for 48 h at 28 °C in a water bath while shaking at 160 rpm. The mycelium was filtered, washed with water, squeezed out and pestled to a fine powder. 200 mg of mycelial powder was resuspended in 1 ml IPP150 buffer (10 mM Tris–HCl pH 8.0, 150 mM NaCl, 0.1% NP40) containing Complete™ Protease Inhibitor Cocktail (Roche). After addition of 500 μl glass beads, the suspension was beaten at 4 °C for 20 min and centrifuged at 13000 rpm for 10 min. The supernatant was taken off, recentrifuged and taken off again. 50 μl magnetic Pan Mouse IgG Dynabeads® were washed three times with 1 ml PBS buffer containing 5 mg/ml BSA and subsequently incubated over-night in PBS/BSA solution containing either 2.5 μg mouse anti-GFP (Roche applied science, mixture of clones 7.1 and 13.1) or 2.5 μg mouse anti-HA antibody (12CA5). Afterwards, the beads were washed three times with PBS/BSA. 350 μl of mycelial raw extract (= input) was added to the beads and incubated for 2 h. The beads were washed 6 times with 1 ml IPP150 containing protease inhibitor. Purified proteins were eluted from the beads by incubation for 7 min at 95 °C in 50 μl 3xSDS sample buffer (300 mM Tris–HCl pH 6.8, 6% SDS, 30% glycerol, 60 μg/ml Bromophenol blue, 10% 2-Mercaptoethanol).

### Mass spectrometric analysis

Mycelium was grown in two replicates as described above for co-immunoprecipitation experiments. 400 mg of mycelial powder was harvested, resuspended in IPP150 buffer containing cOmplete™ Protease Inhibitor and PhosSTOP Phosphatase Inhibitor Cocktails (Roche) and beaten with glass beads for 30 min at 4 °C. After centrifugation at 13000 rpm for 10 min, the supernatant was taken off and purified again by centrifugation. The raw extract was incubated for 2 h with anti-HA antibody covered magnetic beads as described above. The beads were washed 6 times with IPP150 buffer containing protease and phosphatase inhibitor. Purified proteins were eluted from the beads using two times 50 μl of 0.2 M glycine-HCl buffer (pH 2.5) and then neutralized with 10 μl of 1 M Tris–HCl (pH 10.4). Tryptic digestion of the eluted samples, their processing and Nano-LC-MS/MS analysis was performed as described [64]. Raw data files were evaluated using Peaks7 (Bioinformatics Solutions Inc., Waterloo, ON, Canada) and a *Colletotrichum higginsianum* proteome database [26]. The amino acid sequence of Cbk1-HA was added manually to this database. For quantification of peak areas, two groups (*CBK1*-HA vs parental strain) containing the respective two biological replicates were used. The samples were normalized by the total ion count (TIC) and only proteins that were at least 10 times more abundant in the *CBK1*-HA samples with a significance of 10 or more were included in the analysis.

## Additional files

Additional file 1: Figure S1.
*vir-88* produces irregularly shaped appressoria. (PPTX 3922 kb)
Additional file 2: Figure S2. Δ*Chmob1* mutants are sensitive against cell wall stress induced by congo red. (PPTX 5212 kb)
Additional file 3: Text S1 S2. T-DNA flanking sequence of *vir-88* and *ChMOB2* RACE PCR Sequences. (DOCX 15 kb)
Additional file 4: Figure S3. Silencing constructs directed against *ChMOB2* and *ChCbk1* did not lead to significant reduction of the respective transcripts. (PPTX 598 kb)
Additional file 5: Figure S4.
*MOB2*-GFP and *CBK1*-mCherry in locus fusions are functional. (PPTX 9269 kb)
Additional file 6: Table S1. Nano LC-MS/MS identification of proteins co-purified with Cbk1-HA. (DOCX 18 kb)
Additional file 7: Figure S5. The Δ*Chace2* mutant can be complemented by reintroduction of *ChAce2. (PPTX 4167 kb)*

Additional file 8: Figure S6, S7. Potential ChAce2 targets. (PPTX 4272 kb)
Additional file 9: Figure S8, S9. Functions of ChMob1 and ChMob3. (PPTX 19266 kb)
Additional file 10: Figure S10. The RAM and MEN pathways in *Saccharomyces cerevisiae*. (PPTX 49 kb)
Additional file 11: Text S3. Additional accessions, plasmid constructions, strains, plasmids and oligonucleotides. (DOCX 58 kb)
