# Supplementary material for: ChMob2 binds to ChCbk1 and promotes virulence and conidiation of the fungal pathogen Colletotrichum higginsianum
Source: BMC Microbiol. 2017 Jan 19;17:22. doi: 10.1186/s12866-017-0932-7 (PMC5248491; doi:10.1186/s12866-017-0932-7)
Supplement: Additional file 1: Figure S1. — vir-88 produces irregularly shaped appressoria. (PPTX 3922 kb) [file 12866_2017_932_MOESM1_ESM.pptx]

## Slide 1
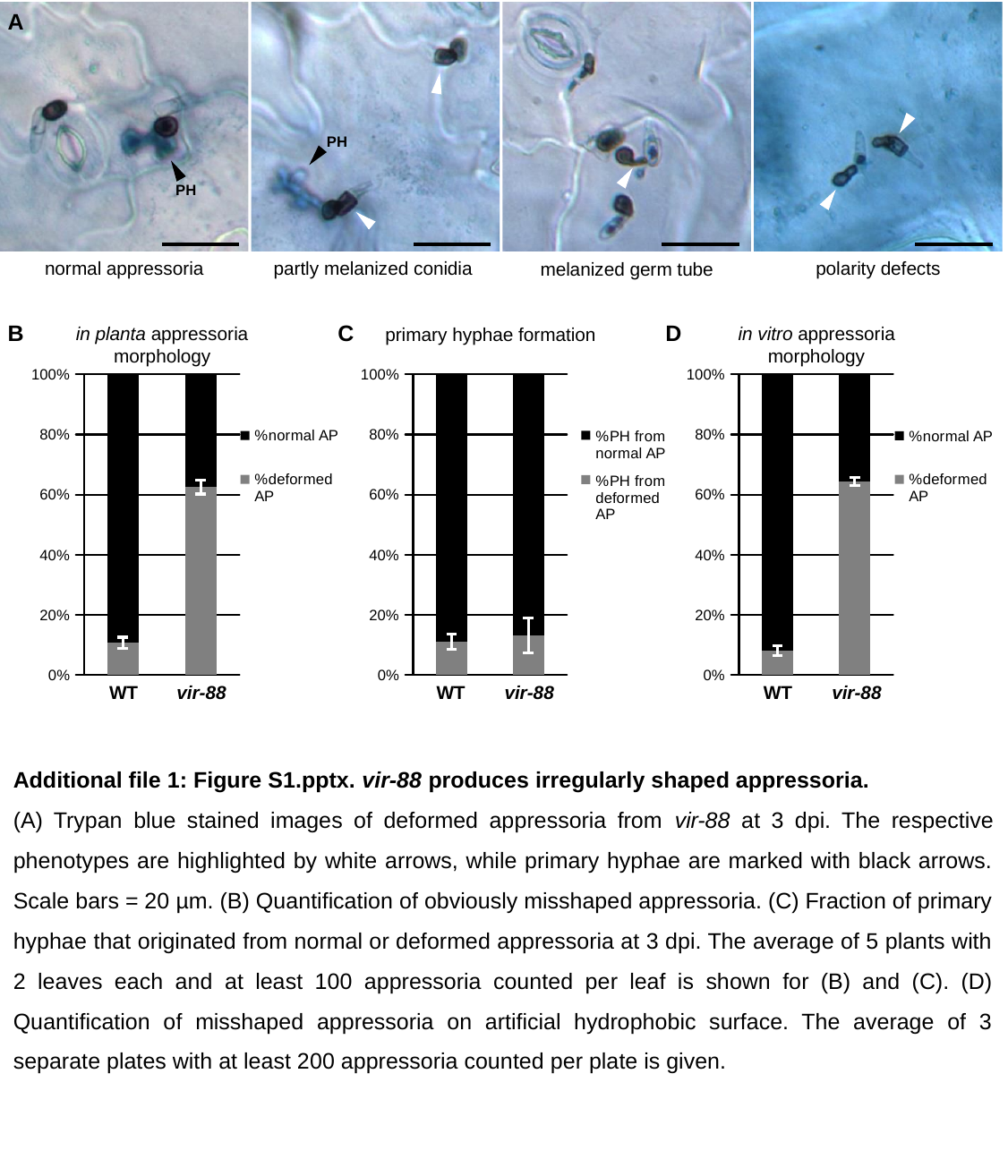

A
PH
PH
polarity defects
normal appressoria
partly melanized conidia
melanized germ tube
B
in planta appressoria morphology
### Chart
| Category | | |
|---|---|---|
| wt | 0.10583375853167498 | 0.894166241468325 |
| vir-88 | 0.6248084933072655 | 0.37519150669273454 |WT
vir-88
C
D
in vitro appressoria morphology
### Chart
| Category | | |
|---|---|---|
| WT | 0.07954545454545454 | 0.9204545454545454 |
| vir-88 | 0.643652561247216 | 0.35634743875278396 |WT
vir-88
primary hyphae formation
### Chart
| Category | | |
|---|---|---|
| | 0.11007072006626846 | 0.8899292799337315 |
| | 0.13062762973352032 | 0.8693723702664797 |WT
vir-88
Additional file 1: Figure S1.pptx. vir-88 produces irregularly shaped appressoria.
(A) Trypan blue stained images of deformed appressoria from vir-88 at 3 dpi. The respective phenotypes are highlighted by white arrows, while primary hyphae are marked with black arrows. Scale bars = 20 µm. (B) Quantification of obviously misshaped appressoria. (C) Fraction of primary hyphae that originated from normal or deformed appressoria at 3 dpi. The average of 5 plants with 2 leaves each and at least 100 appressoria counted per leaf is shown for (B) and (C). (D) Quantification of misshaped appressoria on artificial hydrophobic surface. The average of 3 separate plates with at least 200 appressoria counted per plate is given.
