# Supplementary material for: ChMob2 binds to ChCbk1 and promotes virulence and conidiation of the fungal pathogen Colletotrichum higginsianum
Source: BMC Microbiol. 2017 Jan 19;17:22. doi: 10.1186/s12866-017-0932-7 (PMC5248491; doi:10.1186/s12866-017-0932-7)
Supplement: Additional file 2: Figure S2. — ΔChmob1 mutants are sensitive against cell wall stress induced by congo red. (PPTX 5212 kb) [file 12866_2017_932_MOESM2_ESM.pptx]

## Slide 1
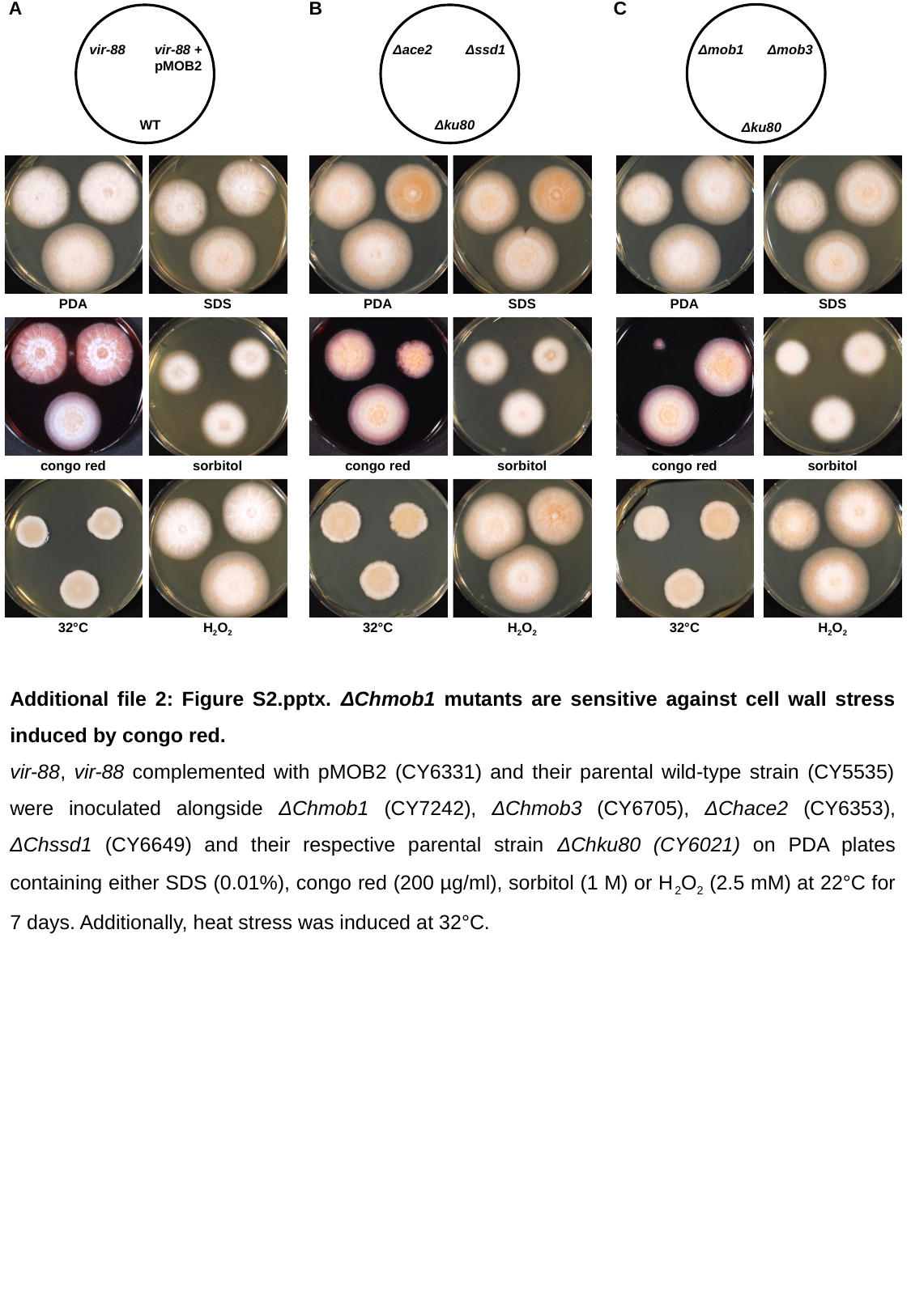

A
B
C
Δmob1
Δmob3
vir-88
vir-88 + pMOB2
Δace2
Δssd1
WT
Δku80
Δku80
SDS
PDA
SDS
PDA
SDS
PDA
congo red
sorbitol
congo red
sorbitol
congo red
sorbitol
32°C
H2O2
32°C
H2O2
32°C
H2O2
Additional file 2: Figure S2.pptx. ΔChmob1 mutants are sensitive against cell wall stress induced by congo red.
vir-88, vir-88 complemented with pMOB2 (CY6331) and their parental wild-type strain (CY5535) were inoculated alongside ΔChmob1 (CY7242), ΔChmob3 (CY6705), ΔChace2 (CY6353), ΔChssd1 (CY6649) and their respective parental strain ΔChku80 (CY6021) on PDA plates containing either SDS (0.01%), congo red (200 µg/ml), sorbitol (1 M) or H2O2 (2.5 mM) at 22°C for 7 days. Additionally, heat stress was induced at 32°C.
