# Supplementary material for: ChMob2 binds to ChCbk1 and promotes virulence and conidiation of the fungal pathogen Colletotrichum higginsianum
Source: BMC Microbiol. 2017 Jan 19;17:22. doi: 10.1186/s12866-017-0932-7 (PMC5248491; doi:10.1186/s12866-017-0932-7)
Supplement: Additional file 3: Text S1 S2. — T-DNA flanking sequence of vir-88 and ChMOB2 RACE PCR Sequences. (DOCX 15 kb) [file 12866_2017_932_MOESM3_ESM.docx]

**Text S1: T-DNA flanking regions of *vir-88***

**Sequence 1: Was obtained by Genome Walker PCR**

template: PvuII digested genomic *vir-88* DNA, ligated with Adaptor

primer CK2575 + CK2583

TATAGGGCNCGCGTGGTCGACGGCCCGGGCTGGTCTGTATCATTGTCTTCCNTGCTCATCCCTTCAGACGCCCTCTTTTGCAGTTTTTGCAAAATTATCCTTCAAATACTNNNTTCCATACACAACTCAAATCCACCAAATCCTTCCCCCTCACTCCGTTTTGCCTTGCGGCCTAGAGGTGCGAGGTCTCAGACATCCCTAGATTGCCAACACAAGCCAGCCCGCAGAGCCATGCCTAGTCTGAGTCAATATCTCTCAACATGGATGGAGCAAGCCCTTGGCTTCTGGTACCGCCCGGCAGCCGGCTTAGTGGTCGTTGTCACCATCACATTAGACATCTTCCTTTGCCTCGGAGTGTTTATTGTGGCCTTGAGGCTCCGTCAGCTAATCAGACATTGATCAACAGCAACGCCCTCAAACACTGATAGTTTAAACTGAAGGCGGGAAACGACAATCTGATCCAAGCTCAAGCTCATTCG

adaptor sequence

supercontig5277 (489-867)

right border (partial)

T-DNA sequence

**Sequence 2: Was obtained by conventional PCR**

template: genomic *vir-88* DNA

primer: CK2711 + CK3135

TTTATCGCCTTGCAGCACATCCCCCTTTCGCCAGCTGGCGTAATAGCGAAGAGGCCCGCACCGATCGCCCTTCCCAACAGTTGCGCAGCCTGAATGGCGAATGAGCTTGAGCTTGGATCAGATTGTCGTTTCCCGCCTTCAGTTTAAACTATCAGTGTTTGAAAGCTGCCAAACCGCCTGGCTCGAACAAGTCCCCGACGTCTTCTACCGCCGGTCAGCAACCTACGTCACCGACCGTTTCCCACGGAAGCCAATCGTCCACCAACCTCGCTCCCAAAGTCCCGCCTCTGCCCAACTCCCCTTCTCTCGCCCACTCTATTGGCATGGACGACCAGGGTAGCTTGACCGACGGAGACAGCATCCTCAACTCCTACCATCTCCCTCGGCCCATGCCTATCTGGCTCAACTCCAACTATGGCAAGCATATTGTCAAGGGCAACTTCATGACCCTCAGCGCGAGACCCAAGACGGTCGAGCAGGGCGAGTGGATCGCTCACCAGGGTAAGTAGCGACTCTATCACTGGTGCCGTTTATCGCTAATGAGACGCAGTCGTCGAGCACTACAGAAACCTCTGGAATTTTGTTCGTGTTCTCCACGAGAAGGAGGAGGATGGCTCGACTATCTGCAATGCCACCACCT

T-DNA sequence

right border (partial)

supercontig5277 (880-1357)

*ChMOB2* sequence (partial)

**Text S2: *ChMOB2* RACE-PCR sequences**

***ChMOB2* 5’-RACE sequence:**

AAGCAGTGGTATCAACGCAGAGTACGCGGGGACAAGCCGCCAAACCGCCTGGCTCGAACAAGTCCCCGACGTCTTCTACCGCCGGTCAGCAACCTACGTCACCGACCGTTTCCCACGGAAGCCAATCGTCCACCAACCTCGCTCCCAAAGTCCCGCCTCTGCCCAACTCCCCTTCTCTCGCCCACTCTATTGGCATGGACGACCAGGGTAGCTTGACCGACGGAGACAGCATCCTCAACTCCTACCATCTCCCTCGG

SMART II A oligo

*ChMOB2* CDS (partial)

*ChMOB2* start codon

**MOB2 3’RACE sequence**

ATGGACGACCAGGGTAGCTTGACCGACGGAGACAGCATCCTCAACTCCTACCATCTCCCTCGGCCCATGCCTATCTGGCTCAACTCCAACTATGGCAAGCATATTGTCAAGGGCAACTTCATGACCCTCAGCGCGAGACCCAAGACGGTCGAGCAGGGCGAGTGGATCGCTCACCAGGTCGTCGAGCACTACAGAAACCTCTGGAATTTTGTTCGTGTTCTCCACGAGAAGGAGGAGGATGGCTCGACTATCTGCAATGCCACCACCTGTCCCAGAATGTCCGCTGGAGCGAACCACTCATTCACCTGGCTCAACAGTCGTCGGGAACCGGTCGAGCTTCCTGCCTTCGAATACATGACTCTTATGCAGAGATGGATCTCGGGCAAGATTGACGACACCAATATCTTCCCGACCGACCCTTCGGGCGTCTCGTATGCGCACAACTCGGCCATCACCACGACGCCTCTCTCGCAACTCACAAACCCCGGCGAGCCCGACTGGATCGGCAAGCGGTCCGGCTTTCCCCAGAACTTTATCGACGTCTGCCAGACCATCTTCCGCCAGATGTTCCGCGTCTACTCCCACCTCTATTGGGCCCATTTTGTCGAGCCCTTCTACCACCTTAACCTGGAGAAGTCTCTCAACAGTTGCTTCAGTCACTTCATCCTGACTGCCACGGCTCTCGACATGCTCAAGCCCCACGAGCTTGAACCCATGCAGCCTTTAATCGACCTCTGGGCTGCCAACGGCACCTTCCCTCCCGAGTCGAAGGCTTACGAGTACGCCAACCTACGAGCTGGCGAGCGCCTGATGCACCTGGCTGGCGTTTCCTAAGTGTATGCGCAAAACTTCACGGCACGGCTAGGAGGATTCAAAGTGGGGTATTACGTTGCAACACGAATTCTGGTGTTCTGTGTCCTTGGGACTTGGGGGTAAAATTCTTGAACACTAGACTCGTCGATCATGTCCCCTTTTTCGCCATGGACGCCGGAACATTCAAGACTGGAAAGAAGTACTAGAGGTTGGGAACCAACACGACAATGGCCCTCCAAAAAAAAAAAAAAAAAAAAAAAAAAGTACTCTGCGTTGATACCACTGCT

*ChMOB2* CDS

*ChMOB2* start codon

*ChMOB2* stop codon

3‘ CDS primer
