# Supplementary material for: ChMob2 binds to ChCbk1 and promotes virulence and conidiation of the fungal pathogen Colletotrichum higginsianum
Source: BMC Microbiol. 2017 Jan 19;17:22. doi: 10.1186/s12866-017-0932-7 (PMC5248491; doi:10.1186/s12866-017-0932-7)
Supplement: Additional file 4: Figure S3. — Silencing constructs directed against ChMOB2 and ChCbk1 did not lead to significant reduction of the respective transcripts. (PPTX 598 kb) [file 12866_2017_932_MOESM4_ESM.pptx]

## Slide 1
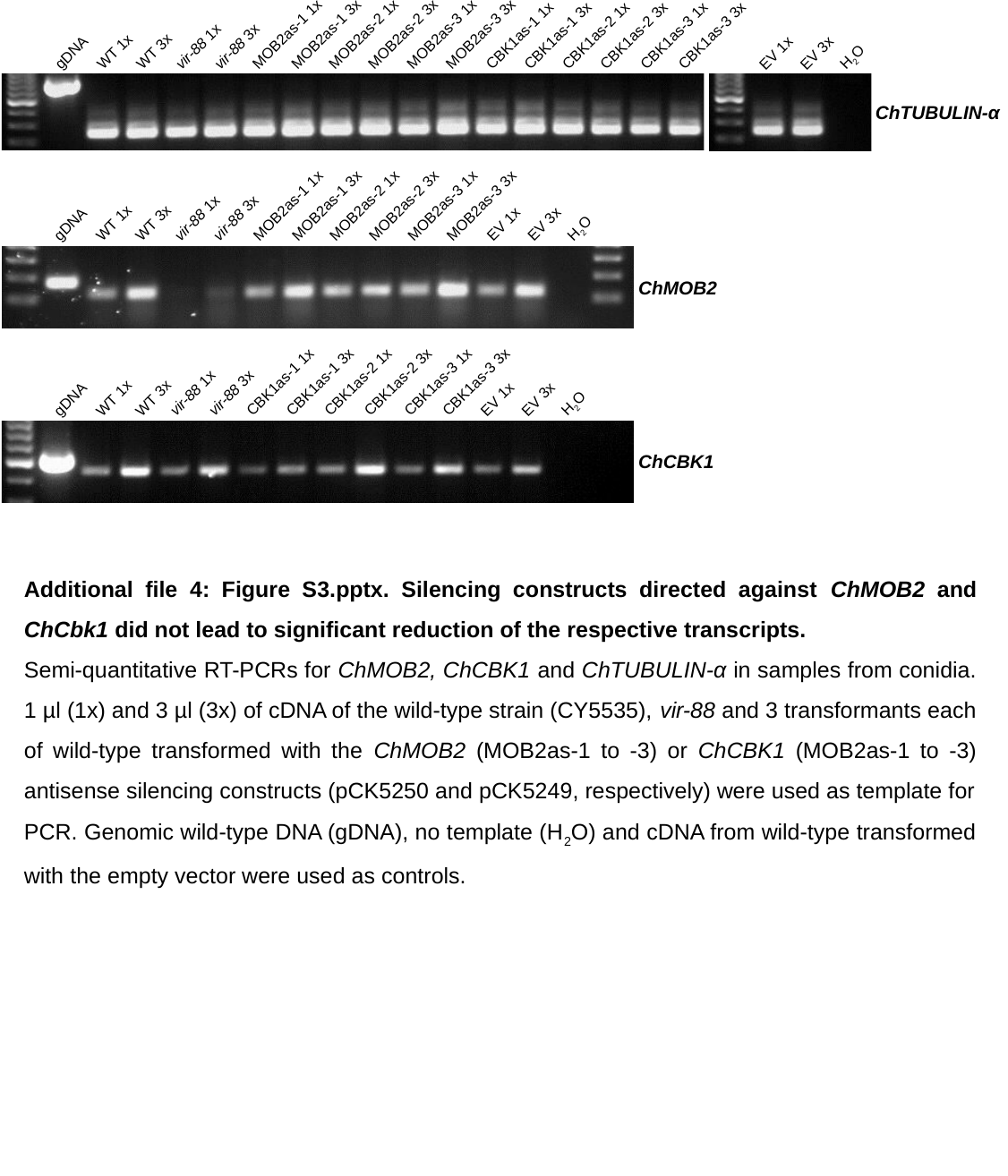

MOB2as-1 1x
MOB2as-1 3x
MOB2as-2 1x
MOB2as-2 3x
MOB2as-3 1x
MOB2as-3 3x
CBK1as-1 1x
CBK1as-1 3x
CBK1as-2 1x
CBK1as-2 3x
CBK1as-3 1x
CBK1as-3 3x
vir-88 1x
vir-88 3x
WT 1x
WT 3x
EV 1x
EV 3x
gDNA
H2O
ChTUBULIN-α
MOB2as-1 1x
MOB2as-1 3x
MOB2as-2 1x
MOB2as-2 3x
MOB2as-3 1x
MOB2as-3 3x
vir-88 1x
vir-88 3x
WT 1x
WT 3x
EV 1x
EV 3x
gDNA
H2O
ChMOB2
CBK1as-1 1x
CBK1as-1 3x
CBK1as-2 1x
CBK1as-2 3x
CBK1as-3 1x
CBK1as-3 3x
vir-88 1x
vir-88 3x
WT 1x
WT 3x
EV 1x
EV 3x
gDNA
H2O
ChCBK1
Additional file 4: Figure S3.pptx. Silencing constructs directed against ChMOB2 and ChCbk1 did not lead to significant reduction of the respective transcripts.
Semi-quantitative RT-PCRs for ChMOB2, ChCBK1 and ChTUBULIN-α in samples from conidia. 1 µl (1x) and 3 µl (3x) of cDNA of the wild-type strain (CY5535), vir-88 and 3 transformants each of wild-type transformed with the ChMOB2 (MOB2as-1 to -3) or ChCBK1 (MOB2as-1 to -3) antisense silencing constructs (pCK5250 and pCK5249, respectively) were used as template for PCR. Genomic wild-type DNA (gDNA), no template (H2O) and cDNA from wild-type transformed with the empty vector were used as controls.
