# Supplementary material for: ChMob2 binds to ChCbk1 and promotes virulence and conidiation of the fungal pathogen Colletotrichum higginsianum
Source: BMC Microbiol. 2017 Jan 19;17:22. doi: 10.1186/s12866-017-0932-7 (PMC5248491; doi:10.1186/s12866-017-0932-7)
Supplement: Additional file 5: Figure S4. — MOB2-GFP and CBK1-mCherry in locus fusions are functional. (PPTX 9269 kb) [file 12866_2017_932_MOESM5_ESM.pptx]

## Slide 1
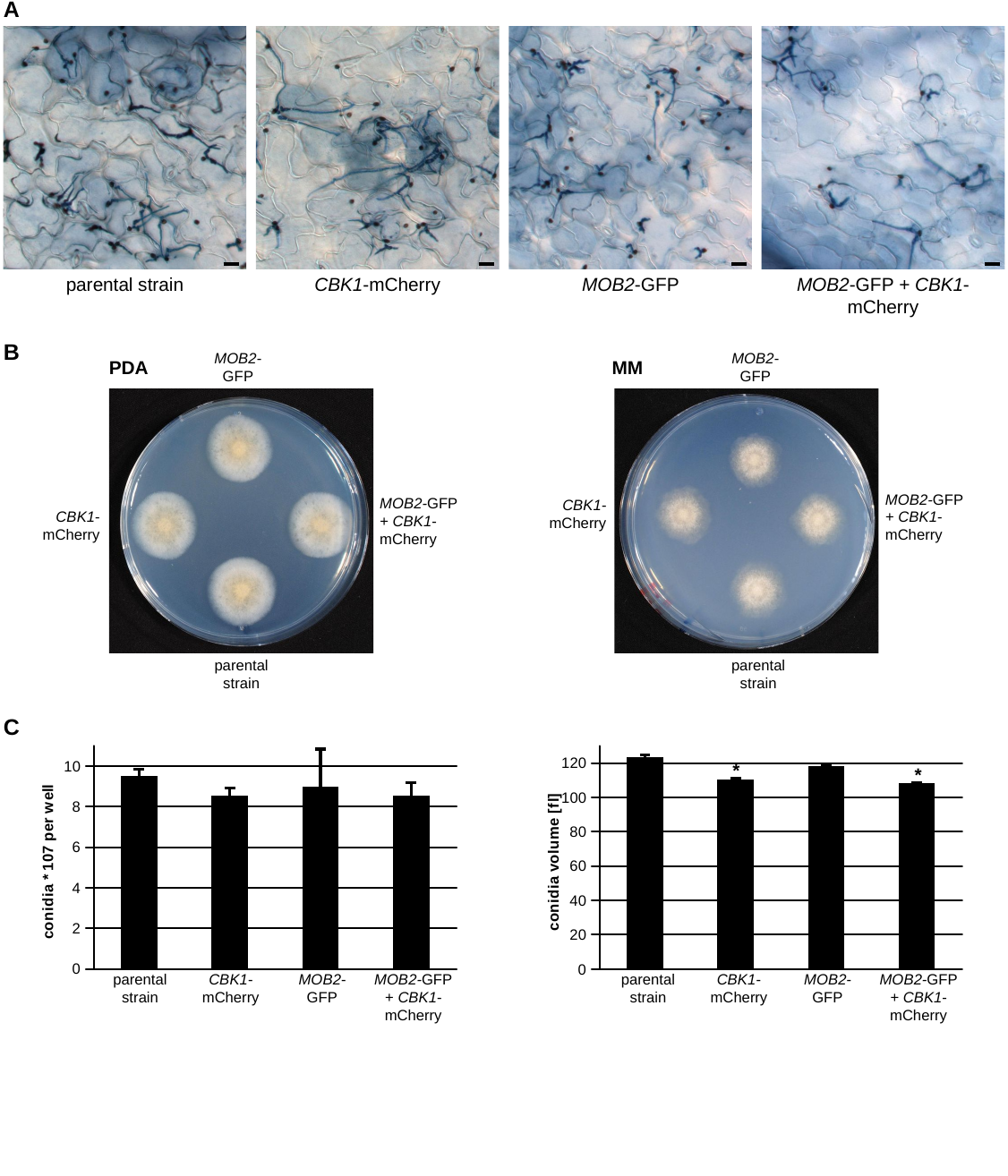

A
CBK1-mCherry
parental strain
MOB2-GFP
MOB2-GFP + CBK1-mCherry
B
MOB2-GFP
PDA
MOB2-GFP + CBK1-mCherry
CBK1-mCherry
parental strain
MOB2-GFP
MM
MOB2-GFP + CBK1-mCherry
CBK1-mCherry
parental strain
C
### Chart
| Category | |
|---|---|
| Δku80 | 9.525 |
| CBK1-mCherry | 8.520000000000001 |
| MOB2-GFP | 8.985 |
| MOB2-GFP + CBK1-mCherry | 8.53 |
### Chart
| Category | |
|---|---|
| Δku80 | 123.7 |
| CBK1-mCherry | 110.60000000000001 |
| MOB2-GFP | 118.13333333333333 |
| MOB2-GFP + CBK1-mCherry | 108.56666666666666 |*
*
parental strain
CBK1-mCherry
MOB2-GFP
MOB2-GFP + CBK1-mCherry
parental strain
CBK1-mCherry
MOB2-GFP
MOB2-GFP + CBK1-mCherry

## Slide 2
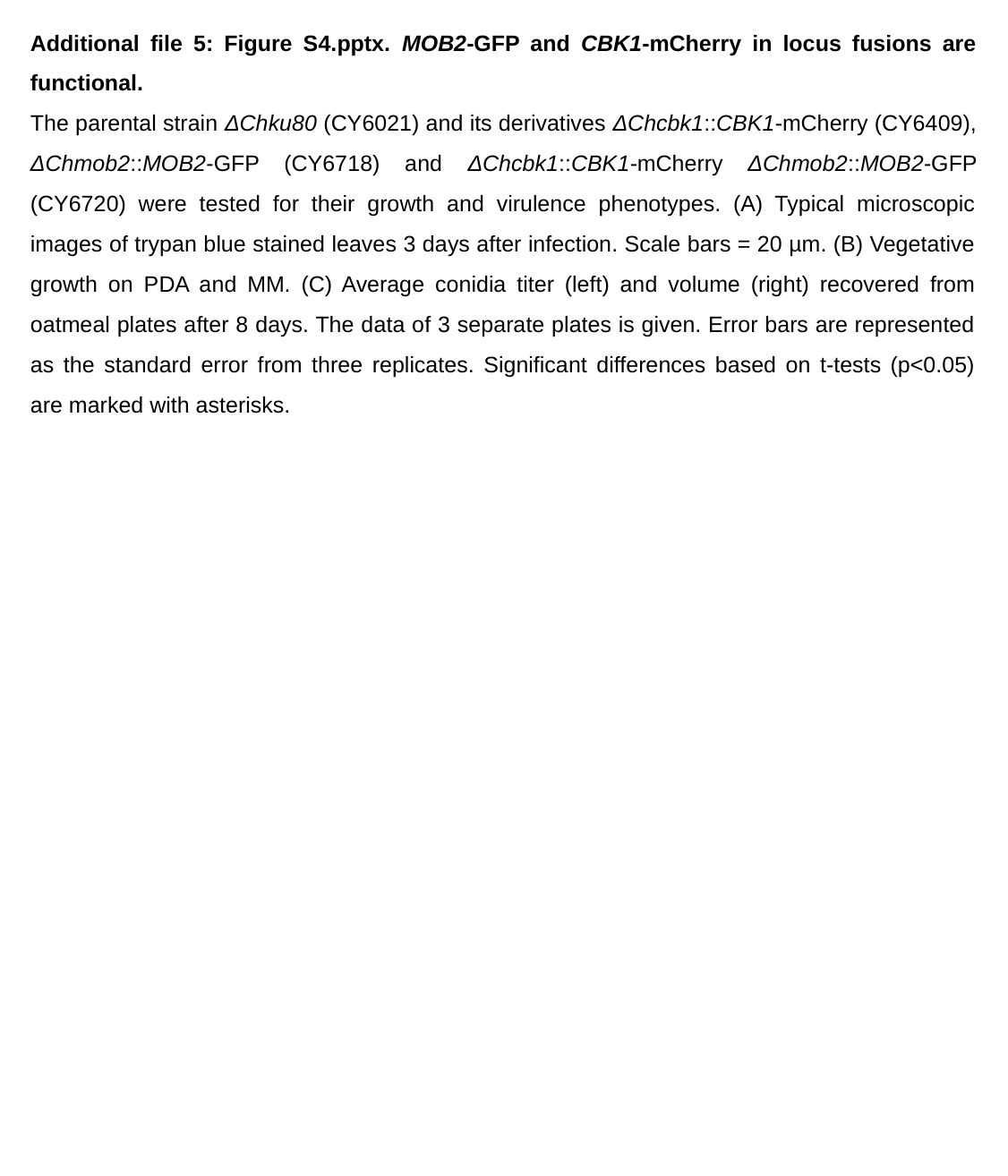

Additional file 5: Figure S4.pptx. MOB2-GFP and CBK1-mCherry in locus fusions are functional.
The parental strain ΔChku80 (CY6021) and its derivatives ΔChcbk1::CBK1-mCherry (CY6409), ΔChmob2::MOB2-GFP (CY6718) and ΔChcbk1::CBK1-mCherry ΔChmob2::MOB2-GFP (CY6720) were tested for their growth and virulence phenotypes. (A) Typical microscopic images of trypan blue stained leaves 3 days after infection. Scale bars = 20 µm. (B) Vegetative growth on PDA and MM. (C) Average conidia titer (left) and volume (right) recovered from oatmeal plates after 8 days. The data of 3 separate plates is given. Error bars are represented as the standard error from three replicates. Significant differences based on t-tests (p<0.05) are marked with asterisks.
