# Supplementary material for: ChMob2 binds to ChCbk1 and promotes virulence and conidiation of the fungal pathogen Colletotrichum higginsianum
Source: BMC Microbiol. 2017 Jan 19;17:22. doi: 10.1186/s12866-017-0932-7 (PMC5248491; doi:10.1186/s12866-017-0932-7)
Supplement: Additional file 6: Table S1. — Nano LC-MS/MS identification of proteins co-purified with Cbk1-HA. (DOCX 18 kb) [file 12866_2017_932_MOESM6_ESM.docx]

### Additional file 6: Table S1.docx. Nano LC-MS/MS identification of proteins co-purified with Cbk1-HA.

| **Significance** | **Coverage (%)** | **# of Peptides** | **Peak area ratio control to Cbk1-HA** | **Description** |
| --- | --- | --- | --- | --- |
| 41.13 | 48 | 31 | 1.00 : 234.13 | CH063_12968 \| serine/threonine-protein kinase cot-1 (661 aa), **“ChCbk1”** |
| 39.53 | 48 | 32 | 1.00 : 232.69 | **Cbk1-HA** |
| 31.67 | 32 | 4 | 0.00 : 1.00 | CH063_09893 \| kinase (199 aa), **N-terminus of** **“ChCbk1”**^1^ |
| 20.90 | 20 | 6 | 1.00 : 14.01 | CH063_02004 \| ATP-dependent RNA helicase eIF4A (397 aa) |
| 17.75 | 23 | 4 | 1.00 : 22.18 | CH063_10798 \| eukaryotic ribosomal protein L18 (184 aa) |
| 16.96 | 29 | 5 | 1.00 : 142.35 | CH063_02865 \| 60S ribosomal protein L7 (258 aa) |
| 16.82 | 25 | 3 | 1.00 : 20.06 | CH063_01772 \| nucleoside diphosphate kinase (150 aa) |
| 16.66 | 33 | 14 | 1.00 : 12.59 | CH063_05296 \| heat shock protein 60 (587 aa) |
| 16.22 | 10 | 7 | 1.00 : 55.73 | CH063_08570 \| phosphoketolase (819 aa) |
| 16.00 | 24 | 3 | 1.00 : 166.07 | CH063_02486 \| 50S ribosomal protein L31e (124 aa) |
| 16.00 | 15 | 3 | 1.00 : 16.05 | CH063_10753 \| ribosomal protein L13e (253 aa) |
| 15.98 | 39 | 5 | 1.00 : 188.16 | CH063_01728 \| 40S ribosomal protein S19 (152 aa) |
| 14.79 | 27 | 8 | 1.00 : 14.01 | CH063_10340 \| phosphoglycerate kinase (419 aa) |
| 14.66 | 12 | 2 | 1.00 : 11.56 | CH063_09218 \| 60S ribosomal protein L11 (174 aa) |
| 14.44 | 45 | 8 | 1.00 : 32.47 | CH063_04712 \| ribosomal protein L6e (203 aa) |
| 14.29 | 29 | 6 | 1.00 : 19.29 | CH063_10312 \| 60S ribosomal protein L10a (218 aa) |
| 14.10 | 50 | 7 | 1.00 : 14.60 | CH063_08215 \| ATP-citrate synthase (209 aa) |
| 13.21 | 15 | 2 | 1.00 : 13.09 | CH063_02636 \| 40S ribosomal protein S13 (152 aa) |
| 12.74 | 42 | 13 | 0.00 : 1.00 | CH063_12012 \| Mob1/phocein family protein (387 aa), **“ChMob2”** |
| 12.64 | 15 | 6 | 1.00 : 36.83 | CH063_01939 \| 6-phosphogluconate dehydrogenase (493 aa) |
| 12.57 | 33 | 7 | 1.00 : 28.72 | CH063_01657 \| guanine nucleotide-binding protein subunit beta-like protein (317 aa) |
| 12.01 | 17 | 2 | 1.00 : 17.64 | CH063_14270 \| 40S ribosomal protein S20 (116 aa) |
| 11.12 | 26 | 4 | 1.00 : 17.54 | CH063_09432 \| 60S ribosomal protein L2 (256 aa) |
| 10.98 | 23 | 4 | 1.00 : 11.10 | CH063_06115 \| 40S ribosomal protein S7 (204 aa) |
| 10.89 | 18 | 5 | 1.00 : 142.45 | CH063_04462 \| pyruvate dehydrogenase E1 component (412 aa) |
| 10.32 | 6 | 2 | 1.00 : 37.72 | CH063_14668 \| 60S ribosomal protein L3 (392 aa) |
| Cutoff values: Significance ≥ 10 and peak area ratio > 10 fold.  ^1^ CH063_09893 is located on a contig overlapping with CH063_12968 and corresponds to the N-terminus of Cbk1. Therefore the N-terminus occurs as an extra, redundant entry in the protein database, while CH063_12968 corresponds to the full length Cbk1 protein. | | | | |
