# Supplementary material for: ChMob2 binds to ChCbk1 and promotes virulence and conidiation of the fungal pathogen Colletotrichum higginsianum
Source: BMC Microbiol. 2017 Jan 19;17:22. doi: 10.1186/s12866-017-0932-7 (PMC5248491; doi:10.1186/s12866-017-0932-7)
Supplement: Additional file 7: Figure S5. — The ΔChace2 mutant can be complemented by reintroduction of ChAce2. (PPTX 4167 kb) [file 12866_2017_932_MOESM7_ESM.pptx]

## Slide 1
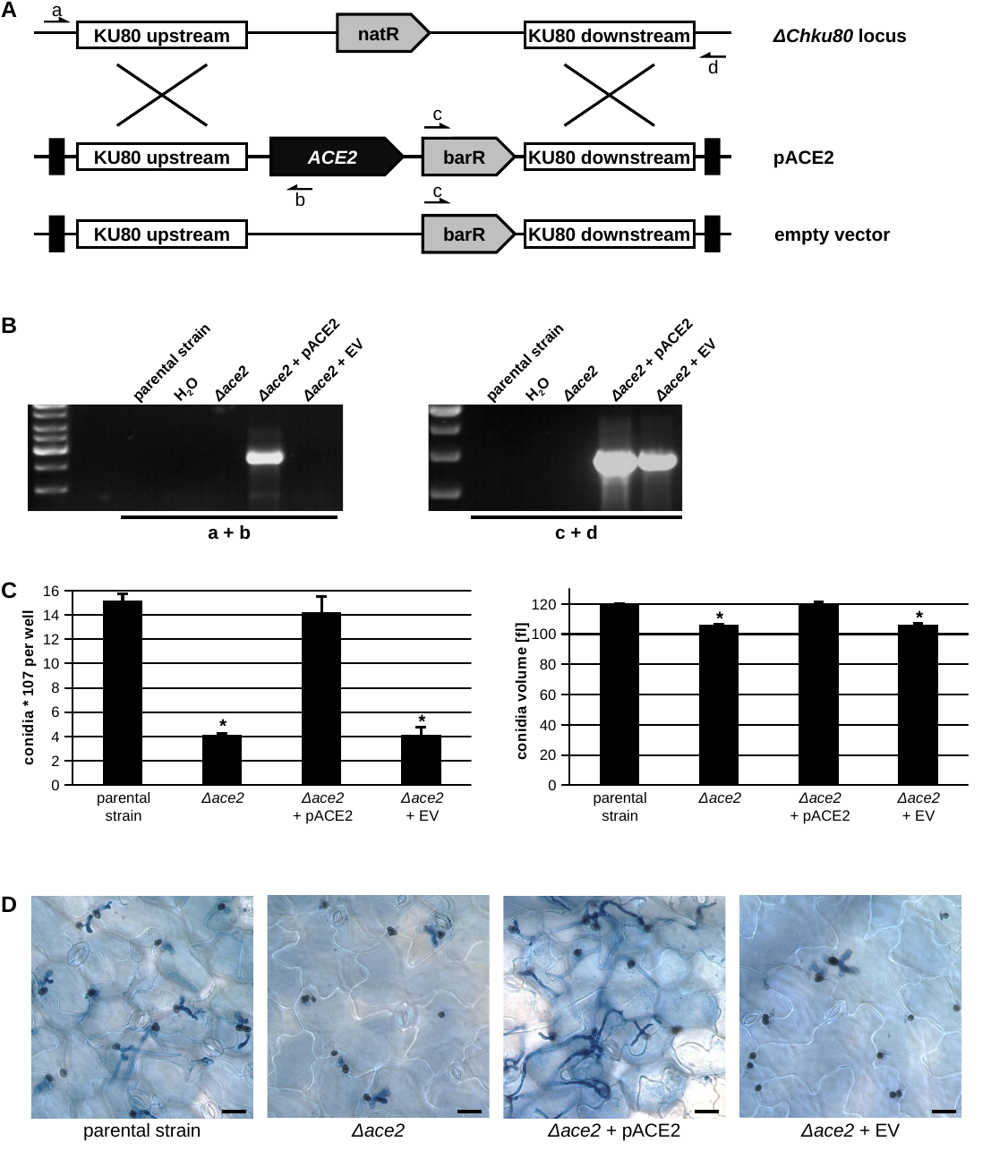

A
a
natR
ΔChku80 locus
KU80 upstream
KU80 downstream
d
c
ACE2
barR
pACE2
KU80 upstream
KU80 downstream
c
b
barR
empty vector
KU80 upstream
KU80 downstream
B
Δace2 + pACE2
Δace2 + pACE2
parental strain
parental strain
Δace2 + EV
Δace2 + EV
Δace2
Δace2
H2O
H2O
c + d
a + b
C
### Chart
| Category | |
|---|---|
| Δku80 | 15.14 |
| Δace2 | 4.159999999999999 |
| Δace2 + pACE2 | 14.226666666666665 |
| Δace2 + EV | 4.16 |
### Chart
| Category | |
|---|---|
| Δku80 | 119.8 |
| Δace2 | 106.30000000000001 |
| Δace2 + pACE2 | 120.46666666666665 |
| Δace2 + EV | 106.09999999999998 |*
*
*
*
parental strain
Δace2
Δace2
+ pACE2
Δace2
+ EV
parental strain
Δace2
Δace2
+ pACE2
Δace2
+ EV
D
parental strain
Δace2
Δace2 + pACE2
Δace2 + EV

## Slide 2
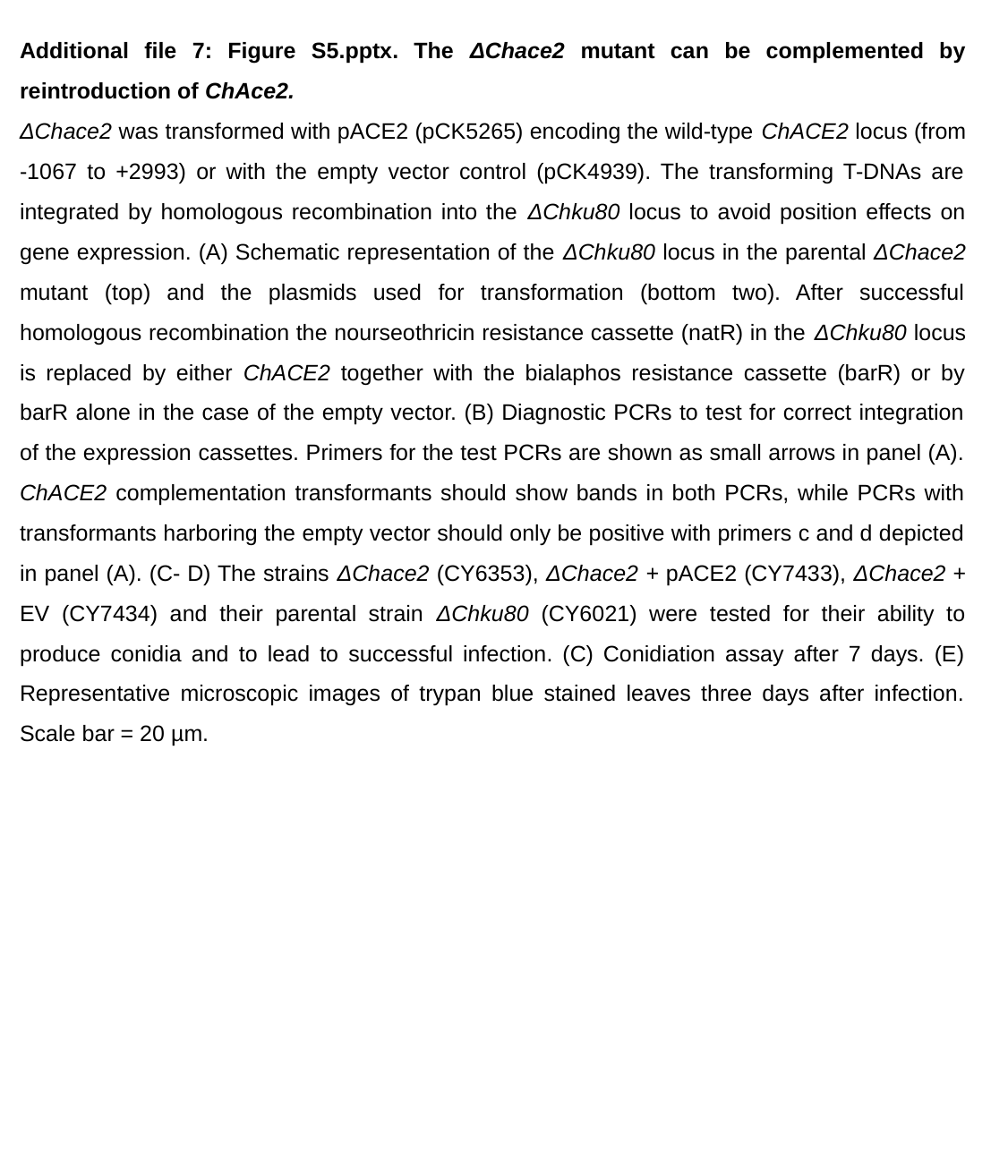

Additional file 7: Figure S5.pptx. The ΔChace2 mutant can be complemented by reintroduction of ChAce2.
ΔChace2 was transformed with pACE2 (pCK5265) encoding the wild-type ChACE2 locus (from -1067 to +2993) or with the empty vector control (pCK4939). The transforming T-DNAs are integrated by homologous recombination into the ΔChku80 locus to avoid position effects on gene expression. (A) Schematic representation of the ΔChku80 locus in the parental ΔChace2 mutant (top) and the plasmids used for transformation (bottom two). After successful homologous recombination the nourseothricin resistance cassette (natR) in the ΔChku80 locus is replaced by either ChACE2 together with the bialaphos resistance cassette (barR) or by barR alone in the case of the empty vector. (B) Diagnostic PCRs to test for correct integration of the expression cassettes. Primers for the test PCRs are shown as small arrows in panel (A). ChACE2 complementation transformants should show bands in both PCRs, while PCRs with transformants harboring the empty vector should only be positive with primers c and d depicted in panel (A). (C- D) The strains ΔChace2 (CY6353), ΔChace2 + pACE2 (CY7433), ΔChace2 + EV (CY7434) and their parental strain ΔChku80 (CY6021) were tested for their ability to produce conidia and to lead to successful infection. (C) Conidiation assay after 7 days. (E) Representative microscopic images of trypan blue stained leaves three days after infection. Scale bar = 20 µm.
