# Supplementary material for: ChMob2 binds to ChCbk1 and promotes virulence and conidiation of the fungal pathogen Colletotrichum higginsianum
Source: BMC Microbiol. 2017 Jan 19;17:22. doi: 10.1186/s12866-017-0932-7 (PMC5248491; doi:10.1186/s12866-017-0932-7)
Supplement: Additional file 8: Figure S6, S7. — Potential ChAce2 targets. (PPTX 4272 kb) [file 12866_2017_932_MOESM8_ESM.pptx]

## Slide 1
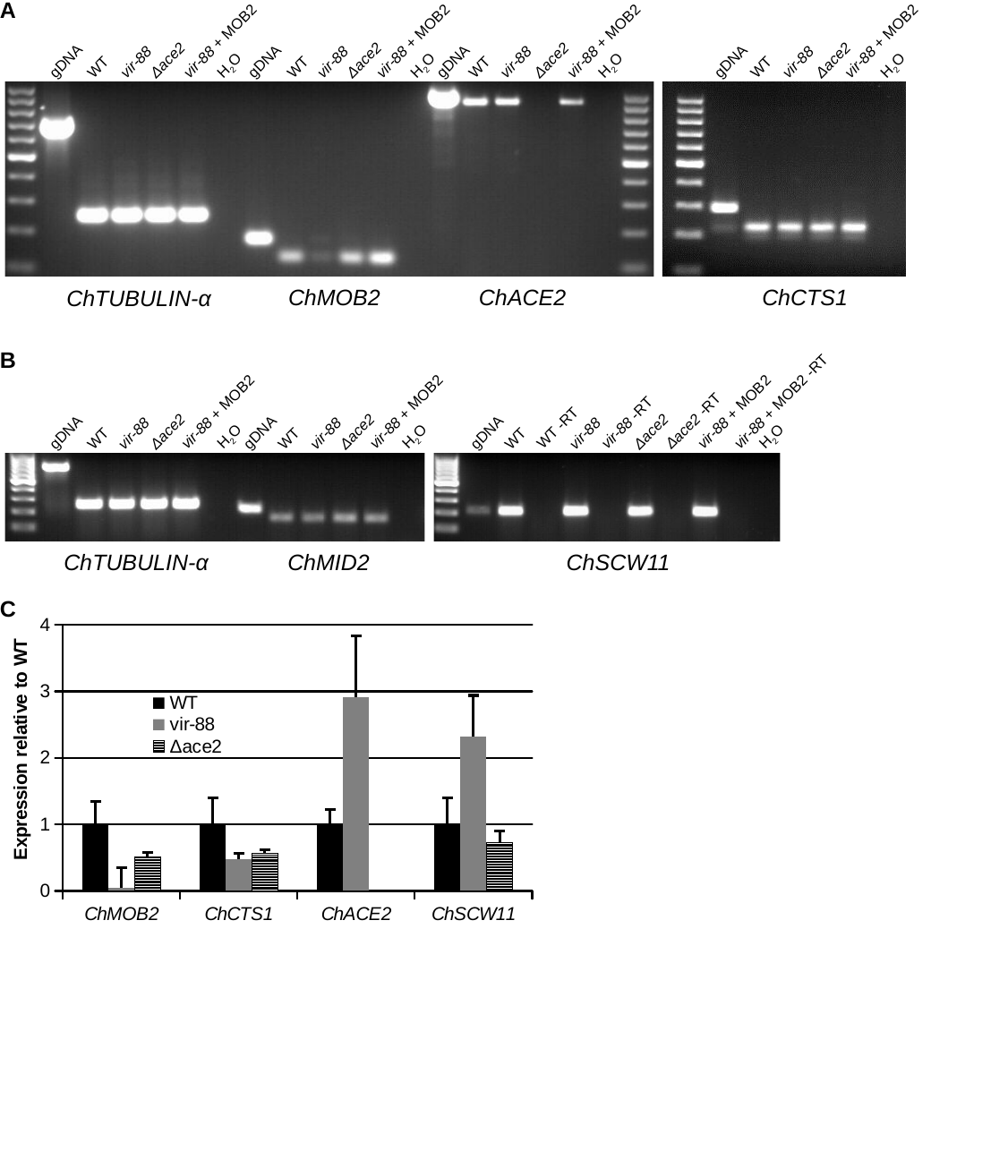

A
vir-88 + MOB2
vir-88 + MOB2
vir-88 + MOB2
vir-88 + MOB2
Δace2
Δace2
Δace2
Δace2
gDNA
gDNA
gDNA
gDNA
vir-88
vir-88
vir-88
vir-88
H2O
H2O
H2O
H2O
WT
WT
WT
WT
ChACE2
ChMOB2
ChCTS1
ChTUBULIN-α
B
vir-88 + MOB2 -RT
vir-88 + MOB2
vir-88 + MOB2
vir-88 + MOB2
Δace2 -RT
vir-88 -RT
WT -RT
Δace2
Δace2
Δace2
gDNA
gDNA
gDNA
vir-88
vir-88
vir-88
H2O
H2O
H2O
WT
WT
WT
ChMID2
ChTUBULIN-α
ChSCW11
C
### Chart
| Category | WT | vir-88 | Δace2 |
|---|---|---|---|
| ChMOB2 | 1.0 | 0.04726554281416016 | 0.5139440368320253 |
| ChCTS1 | 1.0 | 0.48095247769077526 | 0.5578578174568392 |
| ChACE2 | 1.0 | 2.917171473275033 | 0.0 |
| ChSCW11 | 1.0 | 2.326668126259897 | 0.727146154501765 |

## Slide 2
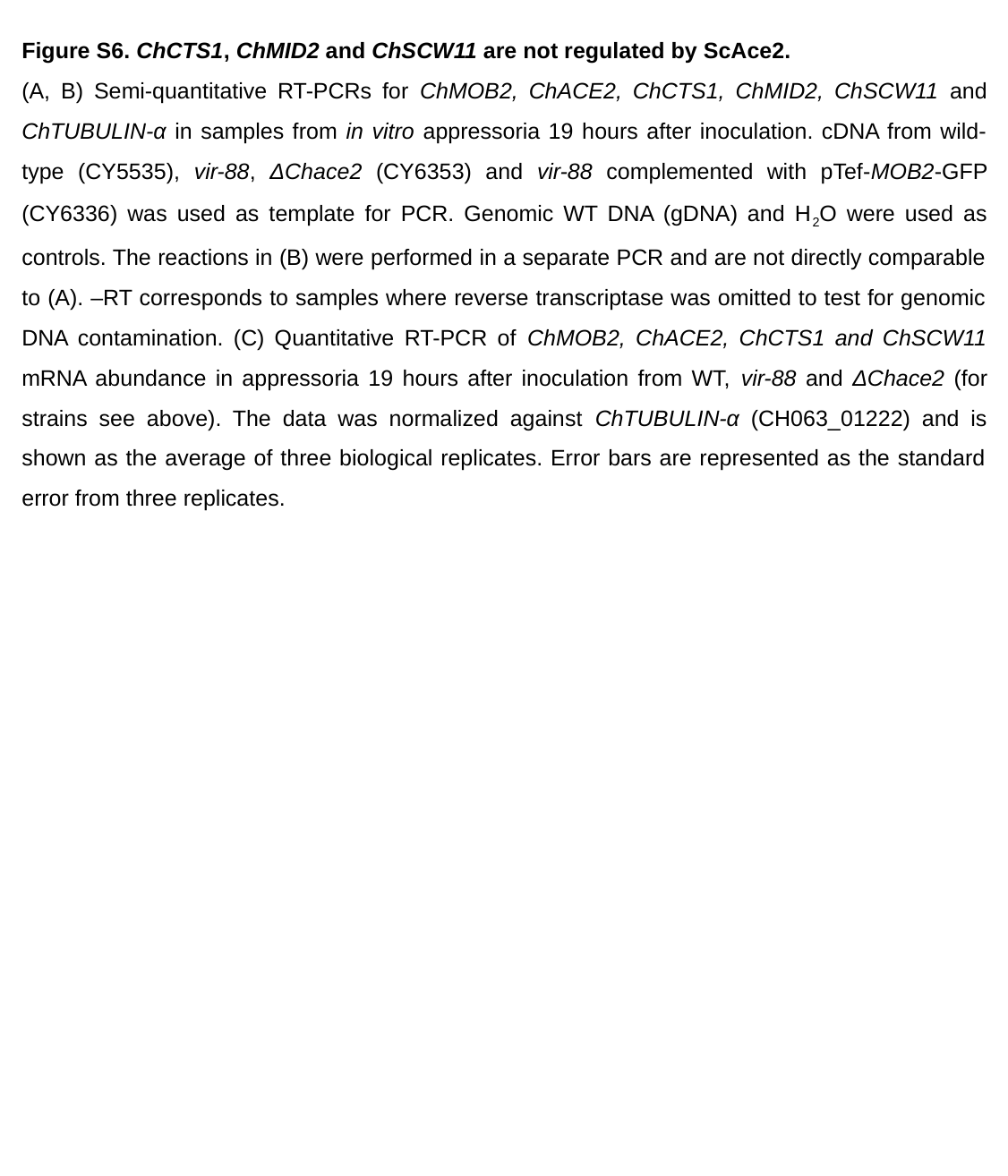

Figure S6. ChCTS1, ChMID2 and ChSCW11 are not regulated by ScAce2.
(A, B) Semi-quantitative RT-PCRs for ChMOB2, ChACE2, ChCTS1, ChMID2, ChSCW11 and ChTUBULIN-α in samples from in vitro appressoria 19 hours after inoculation. cDNA from wild-type (CY5535), vir-88, ΔChace2 (CY6353) and vir-88 complemented with pTef-MOB2-GFP (CY6336) was used as template for PCR. Genomic WT DNA (gDNA) and H2O were used as controls. The reactions in (B) were performed in a separate PCR and are not directly comparable to (A). –RT corresponds to samples where reverse transcriptase was omitted to test for genomic DNA contamination. (C) Quantitative RT-PCR of ChMOB2, ChACE2, ChCTS1 and ChSCW11 mRNA abundance in appressoria 19 hours after inoculation from WT, vir-88 and ΔChace2 (for strains see above). The data was normalized against ChTUBULIN-α (CH063_01222) and is shown as the average of three biological replicates. Error bars are represented as the standard error from three replicates.

## Slide 3
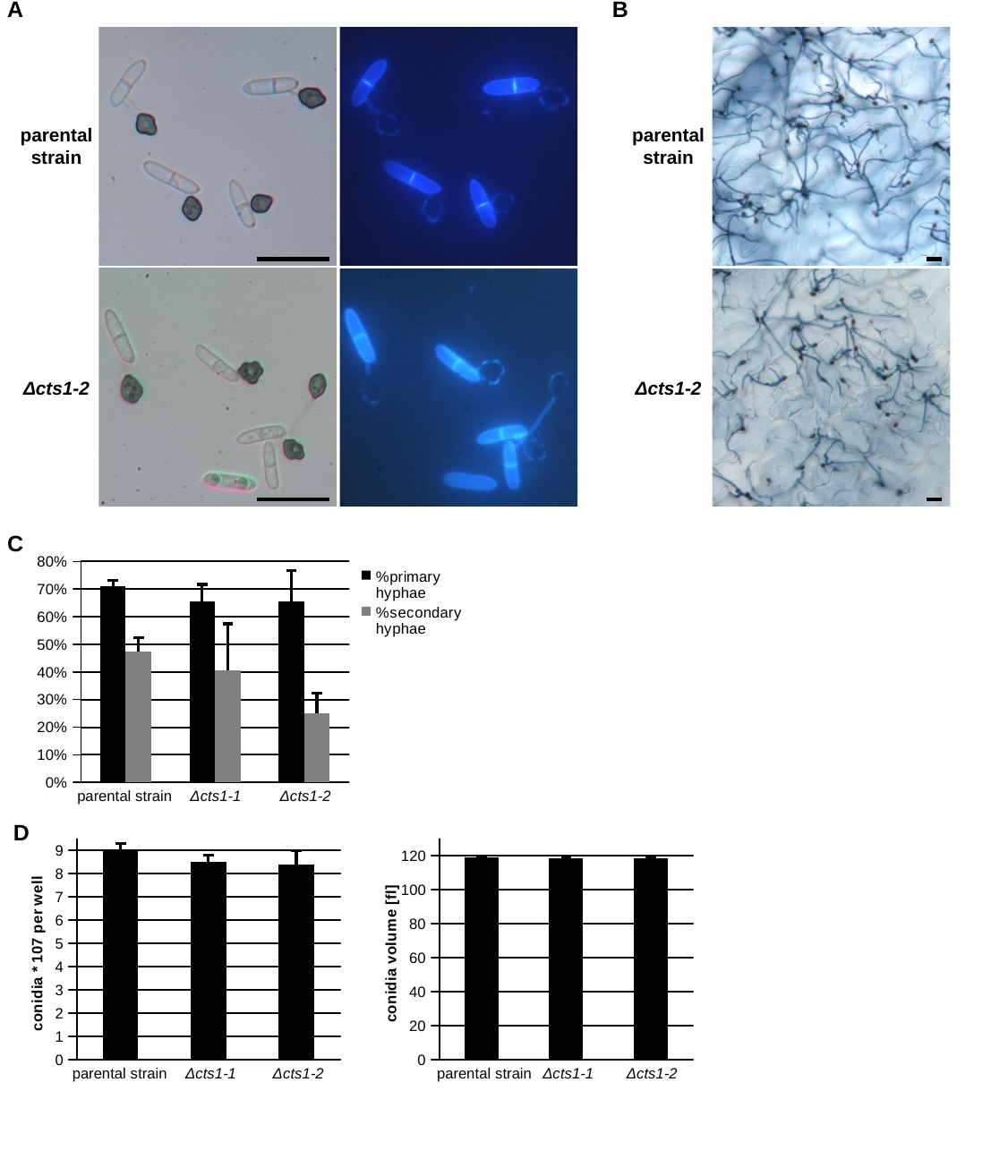

A
B
parental strain
parental strain
Δcts1-2
Δcts1-2
C
### Chart
| Category | | |
|---|---|---|
| Δku80 | 0.7111332877224196 | 0.4745416184767086 |
| Δcts1_1 | 0.6546883835789845 | 0.40427803896927456 |
| Δcts1_2 | 0.6569645904067726 | 0.24961489421278574 |parental strain
Δcts1-1
Δcts1-2
D
### Chart
| Category | |
|---|---|
| ku80 | 9.065 |
| 7110 | 8.51 |
| 7111 | 8.405 |
### Chart
| Category | |
|---|---|
| ku80 | 119.33333333333333 |
| 7110 | 118.7 |
| 7111 | 118.56666666666666 |parental strain
Δcts1-1
Δcts1-2
parental strain
Δcts1-1
Δcts1-2

## Slide 4
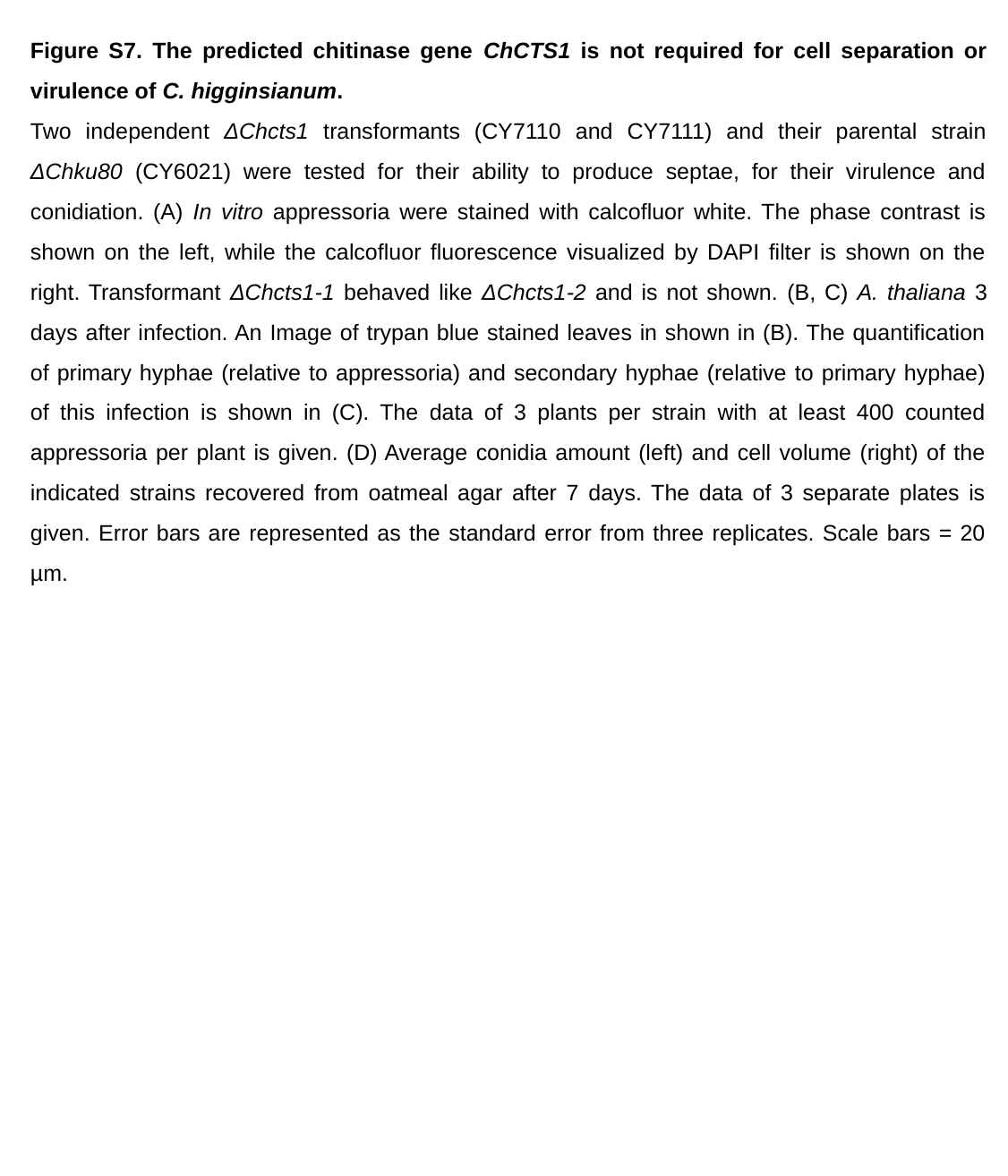

Figure S7. The predicted chitinase gene ChCTS1 is not required for cell separation or virulence of C. higginsianum.
Two independent ΔChcts1 transformants (CY7110 and CY7111) and their parental strain ΔChku80 (CY6021) were tested for their ability to produce septae, for their virulence and conidiation. (A) In vitro appressoria were stained with calcofluor white. The phase contrast is shown on the left, while the calcofluor fluorescence visualized by DAPI filter is shown on the right. Transformant ΔChcts1-1 behaved like ΔChcts1-2 and is not shown. (B, C) A. thaliana 3 days after infection. An Image of trypan blue stained leaves in shown in (B). The quantification of primary hyphae (relative to appressoria) and secondary hyphae (relative to primary hyphae) of this infection is shown in (C). The data of 3 plants per strain with at least 400 counted appressoria per plant is given. (D) Average conidia amount (left) and cell volume (right) of the indicated strains recovered from oatmeal agar after 7 days. The data of 3 separate plates is given. Error bars are represented as the standard error from three replicates. Scale bars = 20 µm.
