# Supplementary material for: ChMob2 binds to ChCbk1 and promotes virulence and conidiation of the fungal pathogen Colletotrichum higginsianum
Source: BMC Microbiol. 2017 Jan 19;17:22. doi: 10.1186/s12866-017-0932-7 (PMC5248491; doi:10.1186/s12866-017-0932-7)
Supplement: Additional file 9: Figure S8, S9. — Functions of ChMob1 and ChMob3. (PPTX 19266 kb) [file 12866_2017_932_MOESM9_ESM.pptx]

## Slide 1
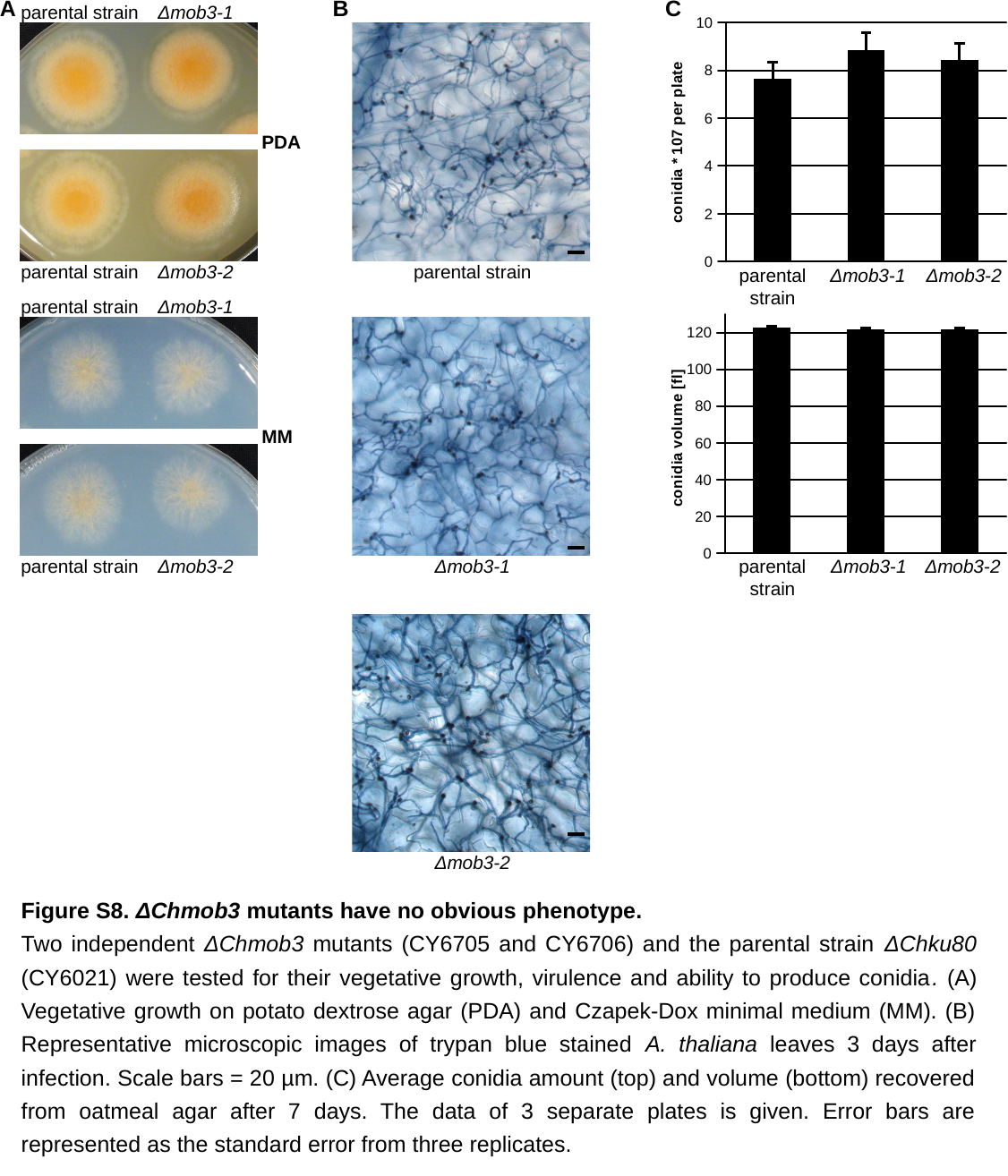

A
B
C
parental strain
Δmob3-1
### Chart
| Category | |
|---|---|
| ∆ku80 | 7.634999999999999 |
| ∆mob3_1 (6705) | 8.834999999999999 |
| ∆mob3_2 (6706) | 8.42 |
PDA
parental strain
Δmob3-2
parental strain
parental
strain
Δmob3-1
Δmob3-2
parental strain
Δmob3-1
### Chart
| Category | |
|---|---|
| ∆ku80 | 122.66666666666667 |
| ∆mob3_1 (6705) | 122.0 |
| ∆mob3_2 (6706) | 122.0 |
MM
parental strain
Δmob3-2
Δmob3-1
parental
strain
Δmob3-1
Δmob3-2
Δmob3-2
Figure S8. ΔChmob3 mutants have no obvious phenotype.
Two independent ΔChmob3 mutants (CY6705 and CY6706) and the parental strain ΔChku80 (CY6021) were tested for their vegetative growth, virulence and ability to produce conidia. (A) Vegetative growth on potato dextrose agar (PDA) and Czapek-Dox minimal medium (MM). (B) Representative microscopic images of trypan blue stained A. thaliana leaves 3 days after infection. Scale bars = 20 µm. (C) Average conidia amount (top) and volume (bottom) recovered from oatmeal agar after 7 days. The data of 3 separate plates is given. Error bars are represented as the standard error from three replicates.

## Slide 2
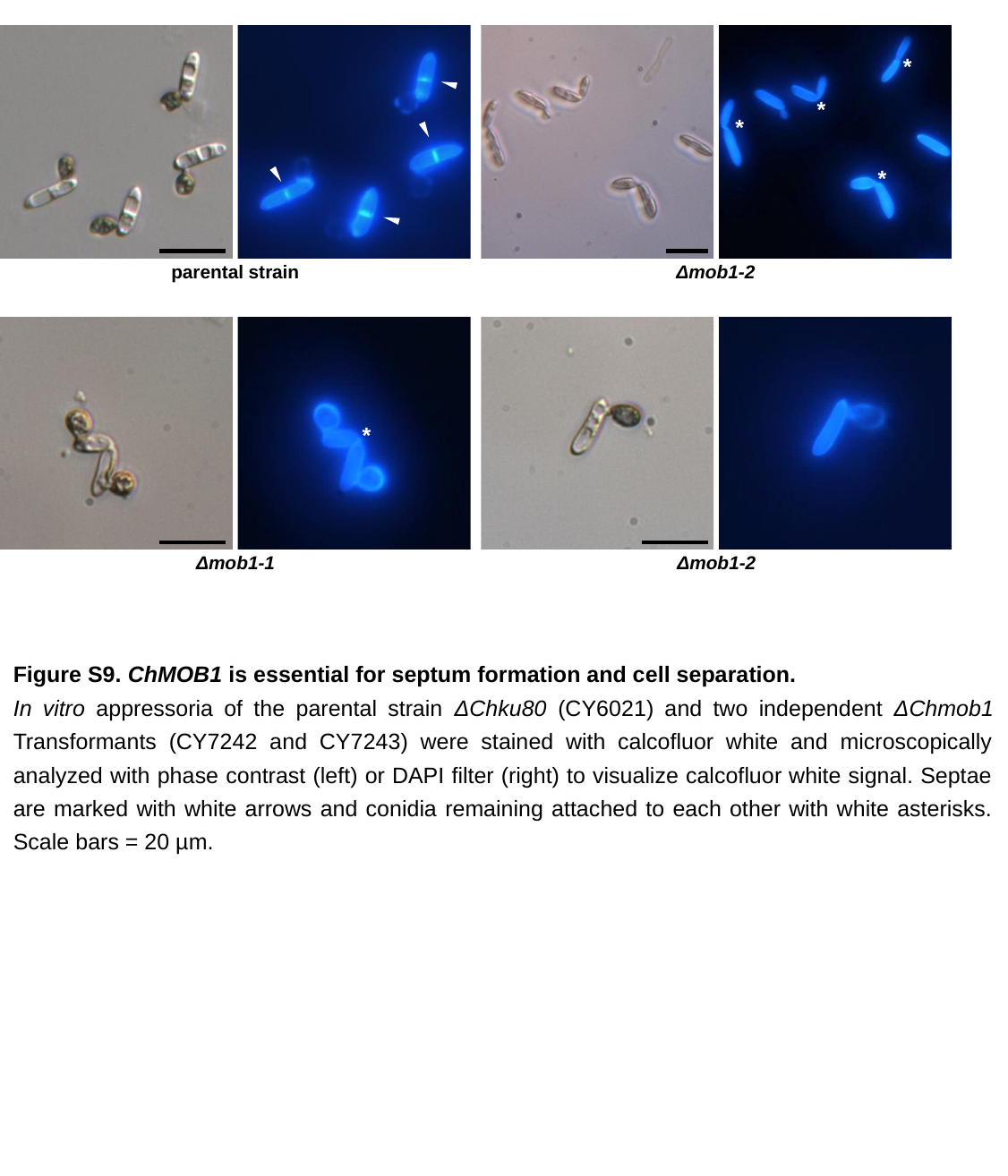

*
*
*
*
parental strain
Δmob1-2
*
Δmob1-1
Δmob1-2
Figure S9. ChMOB1 is essential for septum formation and cell separation.
In vitro appressoria of the parental strain ΔChku80 (CY6021) and two independent ΔChmob1 Transformants (CY7242 and CY7243) were stained with calcofluor white and microscopically analyzed with phase contrast (left) or DAPI filter (right) to visualize calcofluor white signal. Septae are marked with white arrows and conidia remaining attached to each other with white asterisks. Scale bars = 20 µm.
