# Supplementary material for: ChMob2 binds to ChCbk1 and promotes virulence and conidiation of the fungal pathogen Colletotrichum higginsianum
Source: BMC Microbiol. 2017 Jan 19;17:22. doi: 10.1186/s12866-017-0932-7 (PMC5248491; doi:10.1186/s12866-017-0932-7)
Supplement: Additional file 10: Figure S10. — The RAM and MEN pathways in Saccharomyces cerevisiae. (PPTX 49 kb) [file 12866_2017_932_MOESM10_ESM.pptx]

## Slide 1
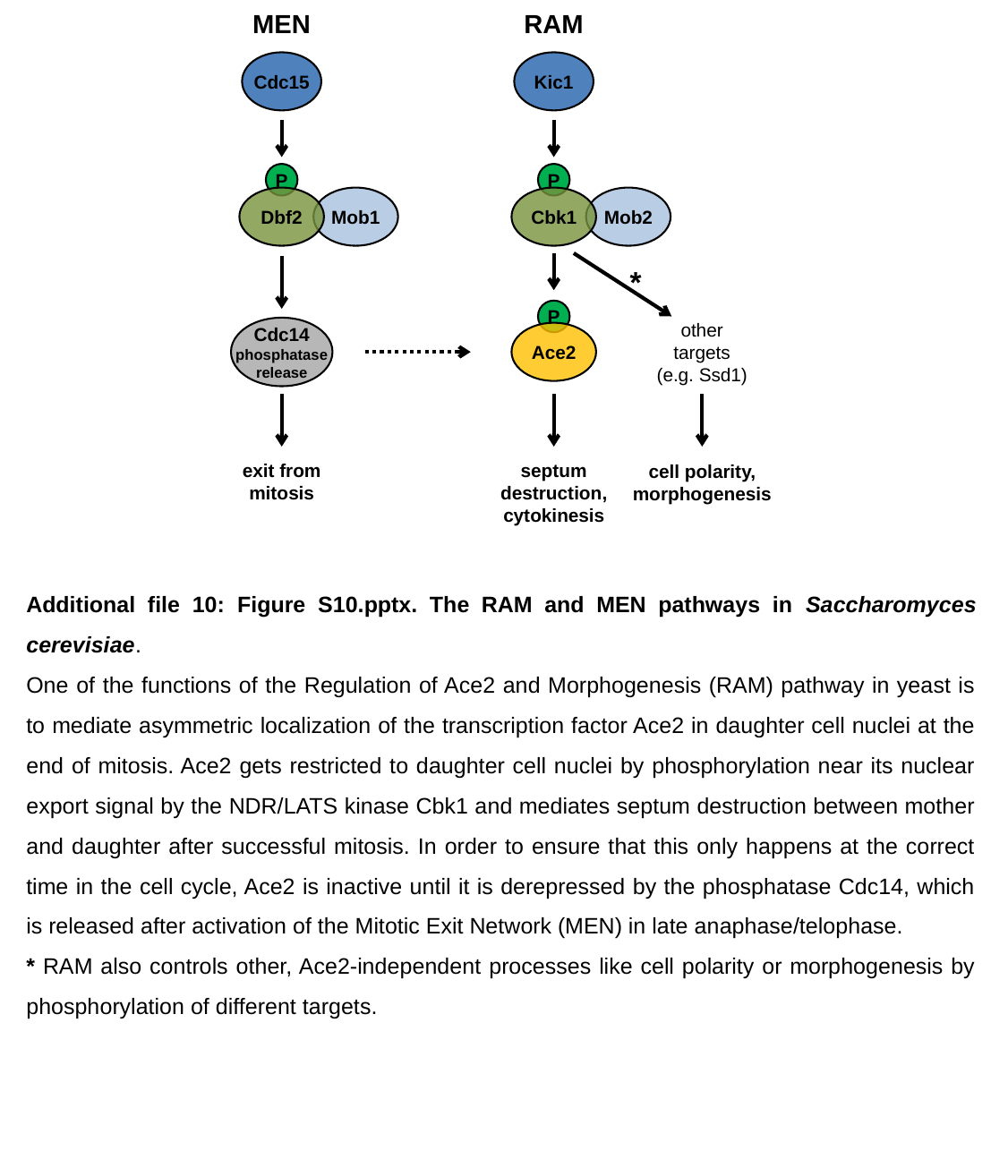

MEN
RAM
Cdc15
Kic1
P
P
Dbf2
Mob1
Cbk1
Mob2
*
P
other targets (e.g. Ssd1)
Cdc14
phosphatase
release
Ace2
exit from mitosis
septum destruction, cytokinesis
cell polarity,
morphogenesis
Additional file 10: Figure S10.pptx. The RAM and MEN pathways in Saccharomyces cerevisiae.
One of the functions of the Regulation of Ace2 and Morphogenesis (RAM) pathway in yeast is to mediate asymmetric localization of the transcription factor Ace2 in daughter cell nuclei at the end of mitosis. Ace2 gets restricted to daughter cell nuclei by phosphorylation near its nuclear export signal by the NDR/LATS kinase Cbk1 and mediates septum destruction between mother and daughter after successful mitosis. In order to ensure that this only happens at the correct time in the cell cycle, Ace2 is inactive until it is derepressed by the phosphatase Cdc14, which is released after activation of the Mitotic Exit Network (MEN) in late anaphase/telophase.
* RAM also controls other, Ace2-independent processes like cell polarity or morphogenesis by phosphorylation of different targets.
