# Supplementary material for: ChMob2 binds to ChCbk1 and promotes virulence and conidiation of the fungal pathogen Colletotrichum higginsianum
Source: BMC Microbiol. 2017 Jan 19;17:22. doi: 10.1186/s12866-017-0932-7 (PMC5248491; doi:10.1186/s12866-017-0932-7)
Supplement: Additional file 11: Text S3. — Additional accessions, plasmid constructions, strains, plasmids and oligonucleotides. (DOCX 58 kb) [file 12866_2017_932_MOESM11_ESM.docx]

**Additional file 11 Text S3.docx: Additional accessions, plasmid constructions, strains, plasmids and oligonucleotides.**

**Additional Genbank accessions:**

| **Organism** | **Mob1** | **Mob2** | **Mob3** |
| --- | --- | --- | --- |
| *Aspergillus fumigatus* | XP_755571 | XP_752284 | XP_755503 |
| *Aspergillus nidulans* | XP_663892 | XP_658974 | XP_663794 |
| *Candida albicans* | XP_719210 | XP_719006 | / |
| *Colletotrichum gloeosporioides* | EQB45785 | EQB44615 | EQB58853 |
| *Colletotrichum graminicola* | XP_008096216 | XP_008090231 | XP_008091793 |
| *Colletotrichum orbiculare* | ENH84350 | ENH78236 | ENH80571 |
| *Fusarium oxysporum f. sp. lycopersici* | EWZ91117 | EWZ91590 | EXA01598 |
| *Magnaporthe oryzae* | XP_003716843 | XP_003709976 | XP_003715285 |
| *Neurospora crassa* | Q9P601 | 2a: NCU03314  2b: NCU07460 | XP_962316 |
| *Saccharomyces cerevisiae* | NP_012160 | NP_116618 | / |
| *Sordaria macrospora* | XP_003350675 | 2a: XP_003345361 2b: XP_003346987 | CBN80575 |
| *Schizosaccharomyces pombe* | NP_595191 | NP_587851 | / |
| *Ustilago maydis* | XP_011391061 | XP_011387502 | XP_011386345 |
| *Verticillium dahliae* | XP_009657797 | XP_009649308 | XP_009650325 |

*Rattus norvegicus* Phocein: NM_133528

**Vectors and plasmid constructions**

pMOB2 (pCK3110) was constructed by cloning a SmaI/StuI digested PCR product amplified with primers CK3092 and CK3093 from wildtype genomic DNA. The resulting product containing the wildtype *ChMOB2* sequence from -1374 to +1797 was cloned into pPN (pCK2650, [[1](#_ENREF_1)]) linearized with PmeI. In the resulting plasmid *ChMOB2* with its promoter region is facing towards the RB of the T-DNA.

pPN-pTef1-GFP (pCK3816) was constructed by first inserting a EcoRV/PmeI digested PCR fragment of the *C. higginsianum* pyruvate kinase polyA site (amplified with primers CK3706 + CK3728) into the PmeI site of pPN (pCK2650) to yield pPN-polyA. Then, a PmeI/NaeI digested GFP fragment amplified from a derivative of pMF280 [[2](#_ENREF_2)] with primers CK3709 + CK3747 was inserted into the PmeI site of the resulting plasmid. Finally, a HpaI/PmeI digested PCR fragment of the *C. higginsianum* translation elongation factor 1 alpha promoter (amplified with primers CK3778 + CK3779) was cloned into the PmeI site of this plasmid to yield pPN-pTef1-GFP. In the resulting plasmid the orientation of the inserted fragments was: Right border - Tef-Promoter – PmeI-site – ATG-GFP- PolyA-site.

pPN-pTef1-MOB2-GFP (pCK4129) was obtained by cloning PmeI digested *ChMOB2* genomic DNA (PCR amplified with primers CK3971 + CK3969) into PmeI linearized pPN-pTef1-GFP (pCK3816). In the resulting plasmid the *ChMOB2* coding region is fused to GFP.

pDelACE2 (pCK4185) for replacement of *ChACE2* with mCherry was generated in two steps. First, a genomic region upstream of *ChACE2* (PCR amplified with primers CK4118 + CK4127) was cloned as a PmeI fragment into the PmeI Site of pBKS-b2r-mCherry-b1r (pCK4122; J. Schmidpeter, in preparation). The resulting plasmid was used for a BP clonase together with pOSCAR, pA-Hyg-OSCAR [[3](#_ENREF_3)] and a genomic region downstream of *ChACE2* (amplified with primers CK4125 + CK4126). The BP clonase reaction was performed as described [[1](#_ENREF_1)].

pINLOCUS-CBK1-mCherry (pCK4270) was generated by cloning the genomic 3’end of *ChCBK1* amplified by PCR with primers CK4199 + CK4200 as a PmeI fragment into the PmeI site of pBKS-b2r-mCherry-b1r (pCK4122; J. Schmidpeter, in preparation). The resulting plasmid was then used in a BP clonase reaction together with pOSCAR, pA-Hyg-OSCAR [[3](#_ENREF_3)] and a genomic region downstream of *ChCBK1* amplified using primers CK4201 + CK4202.

pDelCBK1 (pCK4275) for replacement of *ChCBK1* with mCherry was constructed similar to pDelACE2 (pCK4185) except that the genomic upstream region was amplified with primers CK4203 + CK4204 and the downstream region with CK4201 + CK4202.

pCK4494 was obtained by first cloning a PstI digested PCR fragment containing pTef1-MOB2-GFP-polyA amplified with primers CK4440 + CK4441 using pPN-pTef1-MOB2-GFP (pCK4129) as template into the in-locus vector pA-Bar-OSCAR (pCK3934; J. Schmidpeter, in preparation) which was linearized with PstI. The resulting plasmid was then used in a BP clonase reaction with pOSCAR [[3](#_ENREF_3)] and two regions homologous to contig06386 (amplified with primers CK3666 + CK 3667 and CK3668 + CK3669). This locus (unrelated to *ChMOB2*) was chosen because transformation of *ΔChku80* strains requires homologous regions and previous deletion studies of this region showed no phenotypes.

pINLOCUS-CBK1-HA (pCK4666) was created identical to pINLOCUS-CBK1-mCherry (pCK4270) except that the 3’end of *ChCBK1* was cloned into pBKS-b2r-HA-b1r (pCK4539; J. Schmidpeter, in preparation).

pINLOCUS-MOB2-GFP (pCK4677) was generated by cutting the *ChMOB2* sequence out of pPN-pTef1-MOB2-GFP (pCK4129) using PmeI and cloning the obtained fragment into the PmeI site of pBKS-b2r-GFP-b1r (pCK4387; J. Schmidpeter, in preparation). BP clonase reaction of the resulting plasmid with pOSCAR [[3](#_ENREF_3)], pA-Bar-OSCAR (pCK3934; J. Schmidpeter, in preparation) and a genomic region downstream of *ChMOB2*, which was amplified using primers CK4558 + CK3689, yielded pINLOCUS-MOB2-GFP (pCK4677).

pDelKU80-bar (pCK4939) was generated by BP clonase reaction of *ChKU80* upstream (amplified using CK4932 + CK4933) and downstream (amplified using CK4934 + CK4935) regions together with pA-Bar-OSCAR (pCK3934; J. Schmidpeter, in preparation) and pOSCAR [[3](#_ENREF_3)].

pPN-pTEF-CBK1as (pCK5249) was generated by Gibson Assembly [[4](#_ENREF_4)] of a 787 bp Tef-promoter fragment (amplified with primers CK5223 + CK5224), a 313 bp *ChCBK1*-antisense fragment (reverse complementary to the 3’ end of *ChCBK1*; amplified with CK5225 + CK5226) and pPN-polyA (see construction of pPN-pTef1-GFP (pCK3816)) linearized with MssI.

pPN-pTEF-MOB2as (pCK5250) was generated by Gibson Assembly [[4](#_ENREF_4)] of a 787 bp Tef-promoter PCR fragment (amplified with primers CK5223 + CK5227), a 400 bp *ChMOB2-*antisense PCR fragment (reverse complementary to the 3’ end of *ChMOB2*; amplified with CK5228 + CK5229) and pPN-polyA linearized with MssI.

pACE2 (pCK5265) was generated by cloning a XhoI-digested PCR fragment (amplified with primers CK5221 + CK5222) containing the *ChACE2* gene with 1066 bp of upstream and 482 bp of downstream sequence into the SalI site of pDelKU80-bar (pCK4939).

Plasmids for targeted gene knockouts (except for deletion of *ChCBK1* and *ChACE2*, see above) were all constructed by BP clonase reactions with genomic regions upstream and downstream of the gene of interest together with pOSCAR and pA-Hyg-OSCAR [[3](#_ENREF_3)]. These genomic regions were amplified with the following primers:

*ChMOB1* (pCK4865): CK4846 + CK4847 and CK4848 + CK4849;

*ChMOB2* (pCK3712): CK3686 + CK3687 and CK3688 + CK3689;

*ChMOB3* (pCK4686): CK4626 + CK4627 and CK4628 + CK4629;

*ChSSD1* (pCK4577): CK4568 + CK4569 and CK4566 + CK4567;

*ChCTS1* (pCK4816): CK4778 + CK4779 and CK4780 + CK4781.

**Oligonucleotides**

| Name | Sequence 5’ --> 3’ | Description |
| --- | --- | --- |
| CK2575 | CCTGAATGGCGAATGAGCTTGAGCTT | T-DNA primer for Genome Walker PCR |
| CK2583 | ACTATAGGGCACGCGTGGT | AP2 primer for Genome Walker PCR |
| CK2668 | GCCCTATTCTCGCTCGTCTTCC | *ChTUBULIN-α* RT-PCR primer |
| CK2669 | GGGCTCCAAATCGCAGTAAATG | *ChTUBULIN-α* RT-PCR primer |
| CK2711 | ACCCAACTTAATCGCCTTGCAGCACATC | T-DNA primer |
| CK3092 | gtcctcaaggaaatggtcacg | primer for ampflification of *ChMOB2* wildtype allele |
| CK3093 | gacatggggtgcattatcactt | primer for ampflification of *ChMOB2* wildtype allele |
| CK3135 | CATTCTGGGACAGGTGGTGGCA | primer for amplification of *vir-88* T-DNA flanking region |
| CK3666 | GGGGACAGCTTTCTTGTACAAAGTGGAAGACGACCCCAACGCCGACAG | primer for ampflification of 5’ homology region of contig06386 |
| CK3667 | GGGGACTGCTTTTTTGTACAAACTTGTGGACGCAGAACGCAGGACGC | primer for ampflification of 5’ homology region of contig06386 |
| CK3668 | GGGGACAACTTTGTATAGAAAAGTTGTTTGCAGCGCGAAATAGTCACA | primer for ampflification of 3’ homology region of contig06386 |
| CK3669 | GGGGACAACTTTGTATAATAAAGTTGTACCGACGCTGCGGAAGTTGG | primer for ampflification of 3’ homology region of contig06386 |
| CK3686 | GGGGACAGCTTTCTTGTACAAAGTGGAACAGCAGTCACCATCAATCTTCAGAC | primer for amplification of a *ChMOB2* upstream region |
| CK3687 | GGGGACTGCTTTTTTGTACAAACTTGTGATTAGCTGACGGAGCCTCAAG | primer for amplification of a *ChMOB2* upstream region |
| CK3688 | GGGGACAACTTTGTATAGAAAAGTTGTTCGAGCCCTTCTACCACCTTAACC | primer for amplification of a *ChMOB2* downstream region |
| CK3689 | GGGGACAACTTTGTATAATAAAGTTGTGCTGCGCTTGGATGGTTCATTGT | primer for amplification of a *ChMOB2* downstream region |
| CK3689 | GGGGACAACTTTGTATAATAAAGTTGTGCTGCGCTTGGATGGTTCATTGT | primer for amplification of a *ChMOB2* downstream region |
| CK3706 | GGTTTAAACATGAGCCGCAGTTCGTGGGTGAGT | primer for amplification of pyruvate kinase polyA site |
| CK3728 | GGATATCAGTAAAATTGAATTGTCGGAAGC | primer for amplification of pyruvate kinase polyA site |
| CK3709 | agtaggGTTTAAACATGGTGAGCAAGGGCGAGGAGCTGTT | primer for amplification of GFP CDS |
| CK3710 | atgcgGTTTAAACATGGTGAGCAAGGGCGAGGAGGATA | primer for amplification of mCherry CDS |
| CK3746 | AATGCCGGCTTACTTGTACAGCTCGTCCATGCCG | primer for amplification of mCherry CDS |
| CK3747 | AATGCCGGCTTACTTGTACAGCTCGTCCATGC | primer for amplification of GFP CDS |
| CK3778 | TGGCCCGTTaaCGACCATGCAGACATACCTAGTGT | primer for amplification of Tef1a promoter |
| CK3779 | actgtttaaacTTTGGCGGTTCTGGATCGAGTTGTGTGGT | primer for amplification of Tef1a promoter |
| CK3917 | ATAACTGCAGCTCGAGGTCGACAGAAGATG | primer for amplification of bialaphos resistance casette |
| CK3918 | TTGGATCCTAAATCTCGGTGACGGGCA | primer for amplification of bialaphos resistance casette |
| CK3951 | CTAATACGACTCACTATAGGGCAAGCAGTGGTATCAACGCAGAGT | UPM long, RACE PCR |
| CK3952 | CTAATACGACTCACTATAGGGC | UPM short, RACE PCR |
| CK3959 | ACAACGAGGCCATCTACGAC | *ChTUBULIN-α* qRT-PCR primer |
| CK3960 | GGAGGAAACGACCTGAGCA | *ChTUBULIN-α* qRT-PCR primer, cDNA specific |
| CK3969 | AAGTTTAAACCGGAAACGCCAGCCAGGTG | primer for amplification of *ChMOB2* |
| CK3971 | AAGTTTAAACATGGACGACCAGGGTAGC | primer for amplification of *ChMOB2* |
| CK3983 | AAGCAGTGGTATCAACGCAGAGTACGCGGG | SMART II A oligo, RACE PCR |
| CK4048 | ACTCCCCTTCTCTCGCCCACTCTA | *ChMOB2* RACE PCR |
| CK4049 | CCGAGGGAGATGGTAGGAGTTGAGGATG | *ChMOB2* RACE PCR |
| CK4118 | AAAAGTTTAAACGTGGAGGTCGAAACAAAGATACA | primer for amplification of *ChACE2* upstream region |
| CK4125 | GGGGACAACTTTGTATAGAAAAGTTGTTCAAAGCAGGGTAGTTGATTTCCTTTG | primer for amplification of *ChACE2* downstream region |
| CK4126 | GGGGACAACTTTGTATAATAAAGTTGTCACCGCCTAGTCTTCCGTTCCT | primer for amplification of *ChACE2* downstream region |
| CK4127 | AAAAGTTTAAACTGTGTCGCAAGGTCGAGTC | primer for amplification of *ChACE2* upstream region |
| CK4138 | CGTCAATCTTGCCCGAGA | *ChMOB2* RT-PCR primer |
| CK4144 | ACGGTTTCGACTTCGACCT | *ChCTS1* RT-PCR primer |
| CK4145 | TTGGGGGTATCGGAGGCC | *ChCTS1* RT-PCR primer |
| CK4148 | ACAAGAAGGCAGGACTTTGC | *ChACE2* RT-PCR primer |
| CK4150 | CTGGACATCATCGGAGAGGC | *ChACE2* qRT-PCR and RT-PCR primer |
| CK4183 | GTCCCAGAATGTCCGCTG | *ChMOB2* RT-PCR primer |
| CK4201 | GGGGACAACTTTGTATAGAAAAGTTGTTTATTCGCATCACCCTGGC | primer for amplification of *ChCBK1* downstream region |
| CK4202 | GGGGACAACTTTGTATAATAAAGTTGTGCGAGCTGCGTGGGCTAT | primer for amplification of *ChCBK1* downstream region |
| CK4203 | AAAAGTTTAAACGCCTCCTTGTTTGTCCCTTG | primer for amplification of *ChCBK1* upstream region |
| CK4204 | AAAAGTTTAAACTGTAAAGGTTTCGTCCTGACTC | primer for amplification of *ChCBK1* upstream region |
| CK4440 | AAAACTGCAGTAACGACCATGCAGACATACCT | primer for amplification of pTef1-MOB2-GFP-polyA |
| CK4441 | TATTCTGCAGTCGTTTCCCGCCTTCAGTTTAT | primer for amplification of pTef1-MOB2-GFP-polyA |
| CK4463 | CTGGGAGGGCCACTTGAG | *ChMID2* RT-PCR primer |
| CK4468 | GATGAGACGCTTGGCATTGG | *ChMID2* RT-PCR primer |
| CK4469 | GGAACCTTCGTCAAGAGCCA | *ChSCW11* qRT-PCR and RT-PCR primer |
| CK4470 | CTCCTTCCACTCGTCGTTGT | *ChSCW11* RT-PCR primer |
| CK4502 | TTTTTTTTTTTTTTTTTTTTTTTTTVN | 5’ RACE CDS primer |
| CK4516 | CGAGTTCAAGTACCCCAACCA | *ChCTS1* qRT-PCR primer, cDNA specific |
| CK4517 | TGGAAGTATGTGTCCGAAGTCG | *ChCTS1* qRT-PCR primer |
| CK4520 | CCTCATCAGGATGCAACAAACT | *ChACE2* qRT-PCR primer, cDNA specific |
| CK4522 | CGAGCACTACAGAAACCTCTGG | *ChMOB2* qRT-PCR primer |
| CK4523 | AGGTGAATGAGTGGTTCGCTC | *ChMOB2* qRT-PCR primer, cDNA specific |
| CK4524 | CCTTGCCGTTGCAGTTACC | *ChSCW11* qRT-PCR primer |
| CK4558 | GGGGACAACTTTGTATAGAAAAGTTGTTGTGTATGCGCAAAACTTCACG | primer for amplification of a *ChMOB2* downstream region |
| CK4566 | GGGGACAACTTTGTATAGAAAAGTTGTTAGTGGTGTCTTGATATGTACGGT | primer for amplification of a *ChSSD1* downstream region |
| CK4567 | GGGGACAACTTTGTATAATAAAGTTGTGCAGCACATCTGGGCACTTA | primer for amplification of a *ChSSD1* downstream region |
| CK4568 | GGGGACTGCTTTTTTGTACAAACTTGTAGTCAAGCAGATGAAAGATTGGG | primer for amplification of a *ChSSD1* upstream region |
| CK4569 | GGGGACAGCTTTCTTGTACAAAGTGGAACGCGTCTCCCATCGTACC | primer for amplification of a *ChSSD1* upstream region |
| CK4626 | GGGGACAGCTTTCTTGTACAAAGTGGAACAGAGCAGCAAATGCAACCAT | primer for amplification of a *ChMOB3* upstream region |
| CK4627 | GGGGACTGCTTTTTTGTACAAACTTGTTAAATGGGCGGGCAAGAGAG | primer for amplification of a *ChMOB3* upstream region |
| CK4628 | GGGGACAACTTTGTATAATAAAGTTGTTCGGCAAAACGGATGGGGA | primer for amplification of a *ChMOB3* downstream region |
| CK4629 | GGGGACAACTTTGTATAGAAAAGTTGTTAAGGAGGCATTATTGGGGGAAG | primer for amplification of a *ChMOB3* downstream region |
| CK4778 | GGGGACAGCTTTCTTGTACAAAGTGGAAGGAGAAGGATGACCCCTAATGTGC | primer for amplification of a *ChCTS1* upstream region |
| CK4779 | GGGGACTGCTTTTTTGTACAAACTTGTTGATTGTAAGGTGGCCGAGGC | primer for amplification of a *ChCTS1* upstream region |
| CK4780 | GGGGACAACTTTGTATAGAAAAGTTGTTTGCGGAGGAACGATGATTTATGC | primer for amplification of a *ChCTS1* downstream region |
| CK4781 | GGGGACAACTTTGTATAATAAAGTTGTCGTTGGTTGACCTGAGAAACTTCC | primer for amplification of a *ChCTS1* downstream region |
| CK4846 | GGGGACAGCTTTCTTGTACAAAGTGGAATCTGCACCGAGAGGACCA | primer for amplification of a *ChMOB1* upstream region |
| CK4847 | GGGGACTGCTTTTTTGTACAAACTTGTGGTGTGCGGCAGTTGTTATC | primer for amplification of a *ChMOB1* upstream region |
| CK4848 | GGGGACAACTTTGTATAGAAAAGTTGTTTTGTCTGGGCGTTGGGGC | primer for amplification of a *ChMOB1* downstream region |
| CK4849 | GGGGACAACTTTGTATAATAAAGTTGTAATCCAAACGAAACCCCGGA | primer for amplification of a *ChMOB1* downstream region |
| CK4932 | GGGGACAGCTTTCTTGTACAAAGTGGAATCGAGGACGCGGAACATTGTGA | primer for amplification of a *ChKU80* upstream region |
| CK4933 | GGGGACTGCTTTTTTGTACAAACTTGTCGATGTAGACCGACGCCTCCTT | primer for amplification of a *ChKU80* upstream region |
| CK4934 | GGGGACAACTTTGTATAGAAAAGTTGTTCGACCGACGCGAGATGTGGT | primer for amplification of a *ChKU80* downstream region |
| CK4935 | GGGGACAACTTTGTATAATAAAGTTGTGGATTGTCGATCTTGGACACCAGGA | primer for amplification of a *ChKU80* downstream region |
| CK5221 | TCAGGCTTGGAGGGTCAGCTCG | primer for amplification of *ChACE2* |
| CK5222 | AAAACTCGAGCAAGCAATCACATACGTCGAGGGCA | primer for amplification of *ChACE2* |
| CK5223 | TCGTTTCCCGCCTTCAGTTTGACCATGCAGACATACCTAG | primer for amplification of Tef1a promoter |
| CK5224 | CTTCCGATAGGTTTAAACTTTGGCGGTTCTGGATCG | primer for amplification of Tef1a promoter |
| CK5225 | AACCGCCAAAGTTTAAACCTATCGGAAGTTGTTGTCG | primer for amplification of a *ChCBK1* antisense fragment |
| CK5226 | ACGAACTGCGGCTCATGTTTAAACTCGAGGATCTGCAACACCGAGAAC | primer for amplification of a *ChCBK1* antisense fragment |
| CK5227 | CGTTTCCTAAGTTTAAACTTTGGCGGTTCTGGATCG | primer for amplification of Tef1a promoter |
| CK5228 | AACCGCCAAAGTTTAAACTTAGGAAACGCCAGCCAGG | primer for amplification of a *ChMOB2* antisense fragment |
| CK5229 | ACGAACTGCGGCTCATGTTTAAACTCGAGTGCGCACAACTCGGCCAT | primer for amplification of a *ChMOB2* antisense fragment |
| 3’RACE CDS | AAGCAGTGGTATCAACGCAGAGTAC(T)_30_VN | 3’ RACE CDS primer |
| NUPM | AAGCAGTGGTATCAACGCAGAGT | nested universal primer mix for RACE PCR |

**Plasmids**

| **Name** | **Synonym** | **Description** | **Marker (fungal)** | **Reference** |
| --- | --- | --- | --- | --- |
| pCK2275 | pPK2 | binary plasmid for generation of insertional mutants | hygR | [[5](#_ENREF_5)] |
| pCK2520 | pMF280 | source of GFP |  | [[2](#_ENREF_2)] |
| pCK2549 | g-RB | source of mCherry |  | [[6](#_ENREF_6)] |
| pCK2650 | pPN | binary plasmid; pPK2 derivative | natR | [[1](#_ENREF_1)] |
| pCK3110 | pMOB2 | binary plasmid for complementation of *vir-88* with the wildtype *ChMOB2* allele; pPK2 derivative | natR | this study |
| pCK3272 | pOSCAR | binary vector for multisite gateway reaction |  | [[3](#_ENREF_3)] |
| pCK3273 | pA-Hyg-OSCAR | hygR donor vector for multisite gateway reaction | hygR | [[3](#_ENREF_3)] |
| pCK3712 | pDelMOB2 | binary plasmid for *ChMOB2* deletion; pOSCAR derivative | hygR | this study |
| pCK3806 | pBIG4MRBrev | binary plasmid, source of barR | barR | [[7](#_ENREF_7)] |
| pCK3816 | pPN-pTef1-GFP | binary plasmid for overexpression of c-terminal GFP fusions; pPK2 derivative | natR | this study |
| pCK4129 | pPN-pTef1-MOB2-GFP | binary plasmid for complementation of *vir-88* with pTef1a-MOB2-GFP; pPK2 derivative | natR | this study |
| pCK4185 | pDelACE2 | binary plasmid for replacement of *ChACE2* with mCherry; pOSCAR derivative | hygR | this study |
| pCK4270 | pINLOCUS-CBK1-mCherry | binary plasmid for CBK1-mCherry in locus fusion; pOSCAR derivative | hygR | this study |
| pCK4275 | pDelCBK1 | binary plasmid for replacement of *ChCBK1* with mCherry; pOSCAR derivative | hygR | this study |
| pCK4494 |  | binary plasmid for expression of pTef1a-MOB2-GFP on contig06386 (position 2899 to 3370); pOSCAR derivative | barR | this study |
| pCK4577 | pDelSSD1 | binary plasmid for deletion of *ChSSD1*; pOSCAR derivative | hygR | this study |
| pCK4666 | pINLOCUS-CBK1-HA | binary plasmid for CBK1-6xHA in locus fusion; pOSCAR derivative | hygR | this study |
| pCK4677 | pINLOCUS-MOB2-GFP | binary plasmid for MOB2-GFP in locus fusion; pOSCAR derivative | barR | this study |
| pCK4686 | pDelMOB3 | binary plasmid for deletion of MOB3; pOSCAR derivative | hygR | this study |
| pCK4816 | pDelCTS1 | binary plasmid for deletion of *ChCTS1*; pOSCAR derivative | hygR | this study |
| pCK4865 | pDelMOB1 | binary plasmid for deletion of *ChMOB1*; pOSCAR derivative | hygR | this study |
| pCK4939 | pDelKU80-bar | binary plasmid as empty vector for complementation with *ChACE2*; pOSCAR derivative | barR | this study |
| pCK5249 | pPN-pTEF-CBK1as | binary plasmid for silencing of *ChCBK1; pPK2 derivative* | natR | this study |
| pCK5250 | pPN-pTEF-MOB2as | binary plasmid for silencing of *ChMOB2; pPK2 derivative* | natR | this study |
| pCK5265 | pACE2 | binary plasmid for complementation of *ΔChace2* with *ChACE2*; pOSCAR derivative | barR | this study |

***C. higginsianum* strains**

| **Name** | **Genotype** | **transforming plasmid** | **parental strain** | **Reference** |
| --- | --- | --- | --- | --- |
| CY5535 | *C. higginsianum* MAFF 305635 |  |  | [[8](#_ENREF_8)] |
| CY6021 | *Δku80::natR* | pCK2831 | CY5535 | [[1](#_ENREF_1)] |
| *vir-88* | ATMT random insertional mutant; hypomorphic *ChMOB2* allele | pPK2 | CY5535 | [[1](#_ENREF_1)] |
| CY6284 | *vir-88* + pPN | pCK2650 | *vir-88* | this study |
| CY6331 | *vir-88* + pMOB2 | pCK3110 | *vir-88* | this study |
| CY6332 | *vir-88* + pTef1a-GFP | pCK3816 | *vir-88* | this study |
| CY6336 | *vir-88* + pTef1a-MOB2-GFP | pCK4129 | *vir-88* | this study |
| CY6353 | *Δku80::natR Δace2::mCherry* | pCK4185 | CY6021 | this study |
| CY6409 | *Δku80::natR Δcbk1::CBK1-mCherry* | pCK4270 | CY6021 | this study |
| CY6543 | *Δku80::natR* pTef1a-MOB2-GFP | pCK4494 | CY6021 | this study |
| CY6649 | *Δku80::natR Δssd1::hygR* | pCK4577 | CY6021 | this study |
| CY6678 | *Δku80::natR Δcbk1::CBK1-6xHA* | pCK4666 | CY6021 | this study |
| CY6681 | *Δku80::natR Δcbk1::CBK1-6xHA* pTef1a-MOB2-GFP | pCK4666 | CY6543 | this study |
| CY6705 | *Δku80::natR Δmob3::hygR* | pCK4686 | CY6021 | this study |
| CY6706 | *Δku80::natR Δmob3::hygR* | pCK4686 | CY6021 | this study |
| CY6718 | *Δku80::natR Δmob2::MOB2-GFP* | pCK4677 | CY6021 | this study |
| CY6720 | *Δku80::natR Δcbk1::CBK1-mCherry Δmob2::MOB2-GFP* | pCK4677 | CY6409 | this study |
| CY7110 | *Δku80::natR Δcts1::hygR* | pCK4816 | CY6021 | this study |
| CY7111 | *Δku80::natR Δcts1::hygR* | pCK4816 | CY6021 | this study |
| CY7242 | *Δku80::natR Δmob1::hygR* | pCK4865 | CY6021 | this study |
| CY7243 | *Δku80::natR Δmob1::hygR* | pCK4865 | CY6021 | this study |
| CY7433 | *Δku80::ChACE2-barR Δace2::mCherry* | pCK5265 | CY6353 | this study |
| CY7434 | *Δku80::barR Δace2::mCherry* | pCK4939 | CY6353 | this study |

1. Korn M, Schmidpeter J, Dahl M, Muller S, Voll LM, Koch C. A Genetic Screen for Pathogenicity Genes in the Hemibiotrophic Fungus Colletotrichum higginsianum Identifies the Plasma Membrane Proton Pump Pma2 Required for Host Penetration. PLoS One. 2015;10:e0125960.

2. Freitag M, Hickey PC, Raju NB, Selker EU, Read ND. GFP as a tool to analyze the organization, dynamics and function of nuclei and microtubules in Neurospora crassa. Fungal Genet Biol. 2004;41:897-910.

3. Paz Z, Garcia-Pedrajas MD, Andrews DL, Klosterman SJ, Baeza-Montanez L, Gold SE. One step construction of Agrobacterium-Recombination-ready-plasmids (OSCAR), an efficient and robust tool for ATMT based gene deletion construction in fungi. Fungal Genet Biol. 2011;48:677-84.

4. Gibson DG. Enzymatic assembly of overlapping DNA fragments. Methods Enzymol. 2011;498:349-61.

5. Covert SF, Kapoor P, Lee MH, Briley A, Nairn CJ. Agrobacterium tumefaciens-mediated transformation of Fusarium circinatum. Mycological Research. 2001;105:259-64.

6. Nelson BK, Cai X, Nebenfuhr A. A multicolored set of in vivo organelle markers for co-localization studies in Arabidopsis and other plants. Plant J. 2007;51:1126-36.

7. Fujihara N, Sakaguchi A, Tanaka S, Fujii S, Tsuji G, Shiraishi T, O'Connell R, Kubo Y. Peroxisome biogenesis factor PEX13 is required for appressorium-mediated plant infection by the anthracnose fungus Colletotrichum orbiculare. Mol Plant Microbe Interact. 2010;23:436-45.

8. O'Connell R, Herbert C, Sreenivasaprasad S, Khatib M, Esquerré-Tugayé M-T, Dumas B. A novel Arabidopsis-Colletotrichum pathosystem for the molecular dissection of plant-fungal interactions. Mol Plant Microbe Interact. 2004;17:272-82.
